# Persistent Local Laplacian Prediction of Protein–Ligand Binding Affinities

**DOI:** 10.1021/acs.jcim.6c02460

**Published:** 2026-09-04

**Authors:** Jian Liu, Hongsong Feng

**Affiliations:** † Mathematical Science Research Center, 232838 Chongqing University of Technology, Chongqing 400054, P. R. China; ‡ Department of Mathematics and Statistics, 14727 University of North Carolina at Charlotte, Charlotte, North Carolina 28223, United States

## Abstract

Accurate prediction of protein–ligand binding affinity remains a central challenge in structure-based drug discovery. The effectiveness of machine learning models critically depends on the quality of molecular representations, for which advanced mathematical frameworks provide powerful tools. In this work, we employ a novel mathematical theory, termed the persistent local Laplacian (PLL), to construct molecular descriptors that capture localized geometric and topological features of biomolecular structures. The PLL framework addresses key limitations of traditional topological data analysis methods, such as persistent homology and the persistent Laplacian, which are often insensitive to local structural variations, while maintaining high computational efficiency. The resulting molecular descriptors are integrated with advanced machine learning algorithms to develop accurate predictive models for protein–ligand binding affinity. The proposed models are systematically evaluated on three well-established benchmark datasets, including PDBbind-v2007, PDBbind-v2013, and PDBbind-v2016, demonstrating consistently strong and competitive predictive performance. Computational results show that the PLL-based models outperform existing approaches, highlighting their potential as a powerful tool for drug discovery, protein engineering, and broader applications in science and engineering.

## Graphical abstract

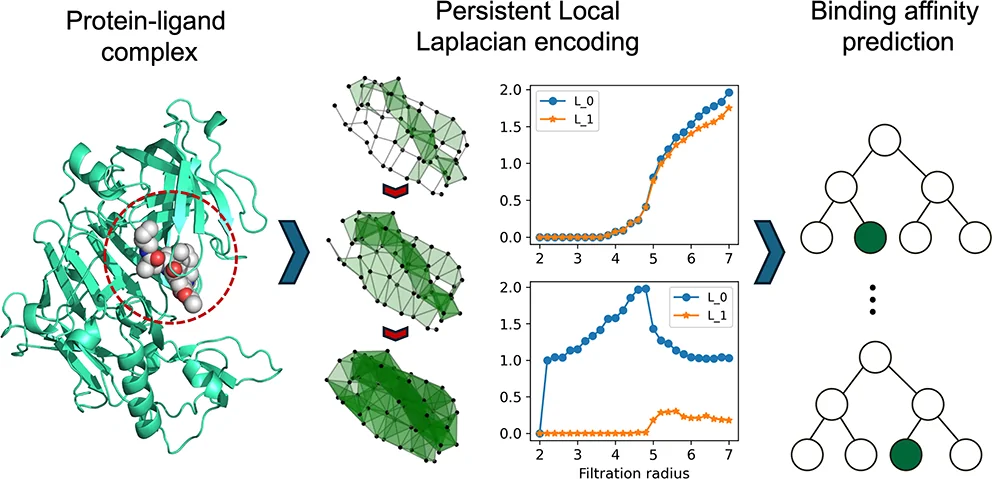

## 1 Introduction

Drug discovery remains time-consuming, costly, and characterized by high failure rates.^1^ Accurate prediction of protein–ligand binding affinity is central to drug design and discovery, as it dictates how small-molecule therapeutics recognize and modulate their biological targets and therapeutic efficacy. Traditional computational approaches, including molecular docking,^2–5^ free energy perturbation,^6^ and empirical scoring functions,^7^ have contributed significantly to structure-based drug design. However, they often suffer from limited accuracy, high computational cost, and difficulty in capturing complex interaction mechanisms, particularly in large-scale virtual screening and the exploration of novel chemical spaces.

Recent advances in machine learning (ML) and artificial intelligence (AI) have transformed drug discovery by enabling data-driven modeling of complex molecular systems.^8–11^ ML models have achieved remarkable success in predicting protein structures, protein–ligand binding affinities, and other key molecular properties by learning nonlinear relationships directly from experimental data.^12,13^ In AI-assisted drug design, molecular descriptors play a critical role in model performance and are widely used in quantitative structure–activity/property relationship analyses and virtual screening pipelines.^14–16^ Because the biological functions of macromolecules depend strongly on their complex three-dimensional (3D) structures and spatial interactions, substantial effort has been devoted to developing 3D structure-based molecular descriptors. Although these descriptors, together with advanced deep learning architectures—such as graph neural networks, convolutional neural networks, and transformer-based models—have improved predictive accuracy,^17–19^ significant challenges remain in effectively capturing essential physicochemical information such as hydrogen bonding, electrostatics, and van der Waals forces, while maintaining a simplified representation of molecular structures.

To address these challenges, advanced mathematical methods grounded in algebraic/geometric topology, spectral theory, and commutative algebra have emerged as powerful tools for biomolecular representation.^20–28^ In particular, topological data analysis methods, such as persistent homology (PH),^29,30^ provide multiscale abstractions of molecular structures that capture essential geometric and topological features. PH has demonstrated strong performance in protein–ligand binding affinity prediction and achieved top-tier results in international drug design competitions such as the D3R Grand Challenges.^31,32^ Beyond the success of PH, persistent combinatorial Laplacian, also known as persistent Laplacian (PL) techniques,^23,33^ offer enhanced molecular representations by encoding additional geometric information through the harmonic and nonharmonic spectra of Laplacian matrices. Several other persistent Laplacian models^22,26–28^ further extend the capabilities of persistent combinatorial Laplacians, thereby enhancing the modeling and analytical power of topological data analysis. Among these methods, the persistent sheaf Laplacian^26^ enables the study of local-to-global properties of structures defined on topological spaces. In particular, it allows a vector space to be assigned to each simplex in a simplicial complex, together with mappings that encode the relationships between these local spaces. The multiscale Gauss linking integral is another recently developed topological method that enables localized characterization of data across multiple spatial scales, with diverse applications in molecular modeling and drug discovery.^25^

As a further advancement, we recently proposed a novel TDA framework, termed persistent local Laplacians (PLL).^34^ While conventional PH and PL methods primarily capture global topological structures and are largely insensitive to local data characteristics, PLL explicitly incorporates localization to characterize both local topological features and geometric information in complex datasets. In particular, PLL enables the simultaneous capture of global topology and local geometric–physical interactions in biomolecular systems, which are crucial for describing the physicochemical interactions that govern molecular functions.

The fundamental idea of PLL lies in investigating the topological and geometric invariants within the neighborhood of a specific point in a dataset. According to the excision theorem in algebraic topology, the local homology group is independent of the global structure of the entire space or dataset.^35^ This key theoretical property implies two significant advantages: first, the computation of local homology and Laplacian only needs to be performed within the neighborhood of a single point, which drastically reduces the computational complexity. Second, the homology and Laplacian calculations at different points are inherently parallelizable, leading to a substantial improvement in the computational efficiency. Another noteworthy advantage of PLL is the scalability of the local feature computation. Specifically, even when the size of the entire dataset expands, the precomputed local topological and geometric information remains unaffected and does not require recalculation. Furthermore, PLL establishes a multiscale detection approach at the local level, which can be analogized to a microscope with adjustable magnification. This unique characteristic enables us to conduct targeted investigations into the information on specific positions within a target object, thereby providing fine-grained insights into local structural properties that were previously difficult to capture with traditional methods.

The objective of this work is to employ PLL theory to develop highly accurate machine learning models for protein–ligand binding affinity prediction. We evaluate our approach on three widely used benchmark datasets from the PDBbind database—PDBbind-v2007, PDBbind-v2013, and PDBbind-v2016.^36^ Given the complexity of these datasets, which involve intricate three-dimensional (3D) protein–ligand structures and diverse physicochemical interactions, we propose element-specific PLL models to effectively capture the underlying interaction patterns in biomolecular systems. As demonstrated in our results, the proposed method outperforms those published models and achieves state-of-the-art performance across these benchmark datasets.

The rest of this paper is organized as follows. Section 2 is devoted to the results of PLLML for protein–ligand binding. Methods are described in Section 3. This paper ends with a conclusion.

## 2 Results

We constructed accurate machine learning models for predicting protein–ligand binding affinity by employing the persistent local Laplacian theory (PLL).^34^ The framework of our models is illustrated in Figure 1.

**Figure 1:**
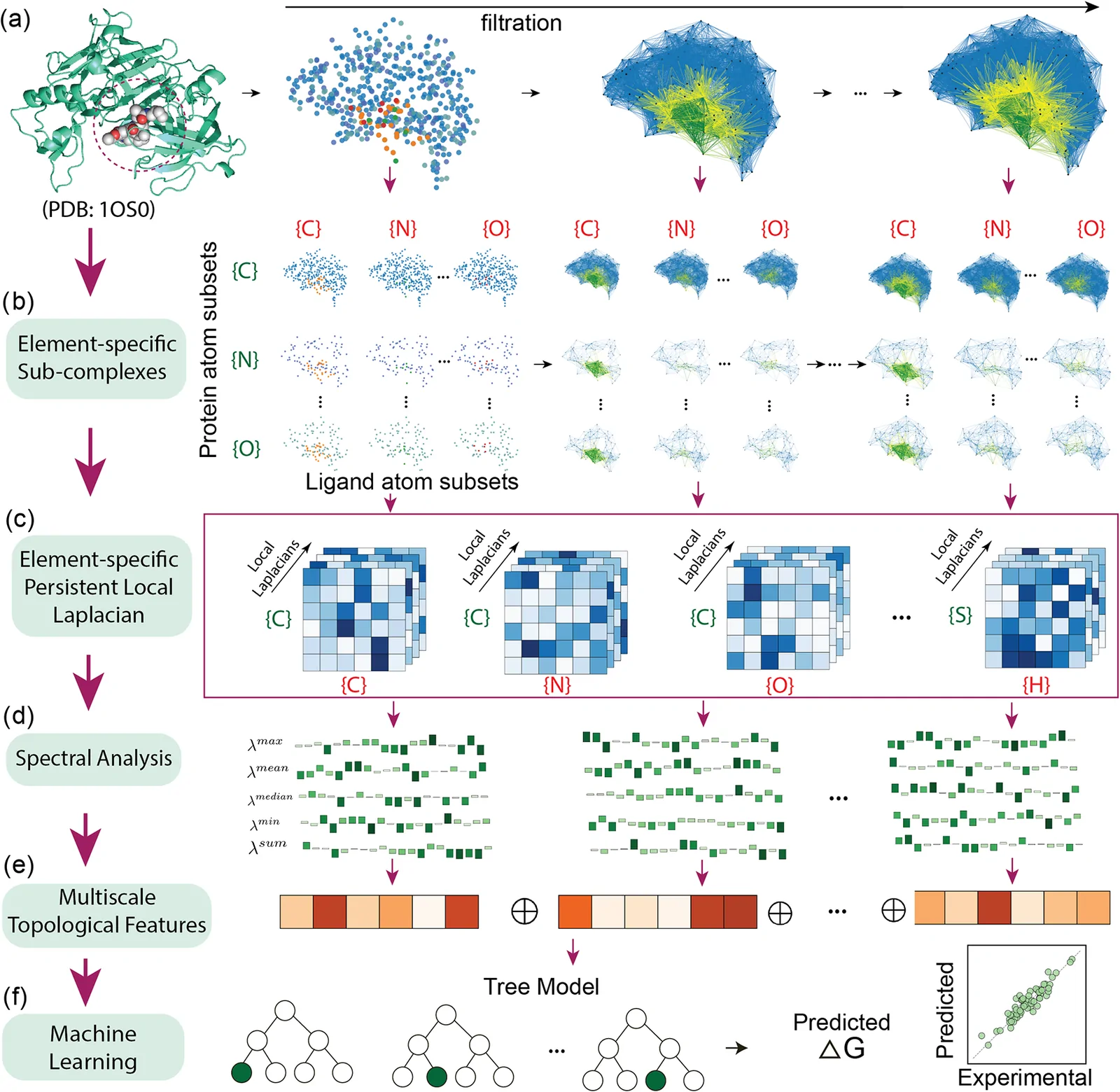
Conceptual diagram of the persistent local Laplacian (PLL) platform for protein–ligand binding affinity prediction. (a) Construction of a Vietoris–Rips complex-induced filtration from atoms in a protein–ligand complex. (b) Generation of nested families of element-specific subcomplexes along distance-based filtration parameters. (c) Formation of atom-specific local Laplacian matrices to characterize atomic interactions within protein–ligand subcomplexes. The ”local Laplacian” arrow indicates the collection of all atom-specific Laplacian matrices for a given atom set. (d) Spectral analysis of the local Laplacian matrices to derive discriminative features for protein–ligand subcomplexes. (e) Multiscale topological features for protein–ligand complexes are established by concatenating features of protein–ligand subcomplexes. (f) Utilization of PLL-based features for downstream binding affinity prediction and analysis using gradient-boosting decision tree models.

### 2.1 Persistent Local Laplacian-Based Molecular Representation

Molecular descriptors are constructed for the protein–ligand complex with PLL, which can also be generalized to other molecular/biomolecular systems. PLL describes three-dimensional structures of molecules while capturing those embedded crucial physicochemical interactions. To this end, we consider element-specific atom grouping on a given protein–ligand complex and represent each atom group with a simplicial complex. A simplicial complex is made up of simplices. A *p*-simplex is the convex hull formed by affinely independent *p* + 1 points. Geometrically, a 0-simplex is a vertex, a 1-simplex is an edge, a 2-simplex is a triangle (with a solid inside region), and a 3-simplex is a solid tetrahedron. A filtration process is applied to give multiscale representations of one atom group, where more edges, triangles, and tetrahedra are created in the simplicial complexes, as shown for representing different protein–ligand atom groups in the Figure 1b. On the basis of these multiscale representations, PLL is then applied to extract intrinsic patterns of simplicial complexes.

Molecules are usually of various sizes, and the corresponding simplicial complexes will have different numbers of simplices along the whole filtration process. To enable scalable characterization, we consider the eigenvalue analysis on a series of Laplacian matrices. Specifically, we put atoms in a molecule into different groups according to their element types or combinations. For instance, we can consider four commonly occurring elements in a protein, including {C, N, O, S}, while nine elements, including {C, N, O, S, P, Cl, Br, I, H}, frequently occur in a ligand. Consequently, considering all pairwise combinations of the protein and ligand elements yields 36 distinct atom groups in a protein–ligand complex. Subsequently, for each atom group and at each filtration scale, we construct a local Laplacian matrix for every atom in the group. Statistical summaries of the eigenvalues of these local Laplacians provide a quantitative description of each atom. These statistical summaries can include the maximum, minimum, mean, standard deviation, and other statistical properties. We then perform further statistical analysis of these eigenvalue-based summaries across all atoms within each atom group, yielding a scalable representation of the corresponding atom group. Finally, spectral analysis across the filtration process provides a comprehensive multiscale description of the protein–ligand complex. A schematic illustration of the molecular descriptor construction is provided in Figure 1a–c. A detailed description of the molecular descriptor construction is provided in the Section 3.

### 2.2 Persistent Local Laplacian Models for Binding Affinity Predictions

Effective molecular descriptors are crucial to the performance of machine learning models. We evaluate the learning capability of the proposed PLL framework through the task of protein–ligand binding affinity prediction, a central challenge in computational drug discovery. The constructed molecular descriptors with PLL are integrated with a gradient boosting decision tree (GBDT) algorithm to build a regression learning model. The modeling pipeline is illustrated in Figure 1a–f. Detailed model configurations are provided in the Section 3. Our experiments and modeling validation utilize the three widely used benchmark datasets from the PDBbind database.^36^ Each dataset provides experimentally measured binding affinities along with high-quality 3D structures of protein–ligand complexes, making them a gold standard for assessing predictive models in this domain.^21,22,24,25,37–39^ Summary statistics for dataset sizes and training–test partitions are reported in Table 3.

We train the models ten times using different random seeds and evaluate their performance using two indicators: the Pearson correlation coefficient (*R*) and the root-mean-square error (RMSE). The predicted binding affinities are compared with the corresponding experimental values. As shown in the first column of Table 1, the average *R* values of the PLL-based machine learning models over the ten runs for the three datasets are 0.813, 0.792, and 0.850, respectively. In addition, the PLL model achieves an average *R* value of 0.818 across the three datasets. These high *R* values demonstrate the effectiveness and reliability of the PLL-based approach for protein–ligand binding affinity prediction. The low RMSE values (in kcal/mol) with an average of 1.873 across the three datasets further confirm the strong predictive performance of our models.

**Table 1:** Modeling Performance of Different Strategies on the Test Sets of the Three PDBbind Benchmark Datasets^a^

dataset	PLL	transformer	PLL + transformer
PDBbind-v2007	0.813 (1.971)	0.785 (2.006)	0.827 (1.925)
PDBbind-v2013	0.792 (1.976)	0.791 (1.974)	0.813 (1.932)
PDBbind-v2016	0.850 (1.672)	0.836 (1.713)	0.861 (1.646)
average	0.818 (1.873)	0.804 (1.898)	0.834 (1.834)

a The evaluation indicators are the Pearson correlation coefficient (*R*) and the root-mean-square error (RMSE, in kcal/mol, in the parentheses). Each model was trained and evaluated over ten independent runs using different random seeds, and the reported results are the averaged metric values. PLL denotes the persistent local Laplacian-based machine learning models. Transformer refers to sequence-based models using natural language processing. PLL + transformer represents the consensus predictions obtained by averaging the outputs of the PLL and transformer models, which is used as our final model, referred to as PLLML.

To further enhance predictive performance, we incorporate natural language processing (NLP)-based molecular features to develop an additional set of machine learning models. Pretrained NLP models generate molecular representations directly from molecular sequences. Specifically, we extract molecular features from protein amino acid sequences using a protein language model^40^ and from small-molecule SMILES strings using a small-molecule language model.^41^ The details of these two NLP models are provided in Section 5 in the Supporting Information. These sequence-derived features are concatenated and combined with the gradient boosting decision tree (GBDT) algorithm to build transformer-based models, whose performance is reported in the second column of Table 1. The average Pearson *R* value across the three datasets is 0.804. In comparison, PLL-based models achieve better performance than the Transformer-based models.

Both sequence-based and 3D structure-based models offer distinct strengths, and we leverage their complementary advantages to further enhance predictive performance. We generate new predictions by averaging the outputs of the PLL and transformer models, resulting in a consensus model referred to as PLLML. The final column of Table 1 reports the performance of this consensus approach. The integrated models have substantially improved accuracy, achieving an average Pearson *R* value of 0.834 across the three datasets. PLLML’s reliability is further demonstrated by the strong alignment between experimental and predicted binding affinities, as visualized in Figure 3.

Our PLLML models outperform existing state-of-the-art approaches, achieving superior predictive accuracy in binding affinity estimation as shown in Figure 2. On the PDBbind-v2016 test set, our PLL model achieves a Pearson *R* value of 0.850 without the boost from the transformer model, which is superior to most published machine learning models. Numerous competitive models grounded in mathematical and physical principles,^14,15,43^ including those based on persistent homology^42^ and persistent spectral theories,^21,22^ have been developed and consistently rank among the strongest performers in this field (see Figure 2). On the PDBbind-v2013 test set, our PLLML model achieved a Pearson *R* value of 0.813, outperforming the persistent spectral theory-based models PerSpect-ML (*R* = 0.793)^21^ and PPS-ML (*R* = 0.791).^22^ On the PDBbind-v2016 test set, our PLLML model achieved a Pearson *R* of 0.861, outperforming the persistent homology-based TopBP-ML (*R* = 0.848)^42^ and the persistent spectral theory-based models PerSpect-ML (*R* = 0.840)^21^ and PPS-ML (*R* = 0.843).^22^ Among these mathematical models, PerSpect-ML achieves the best performance, with *R* = 0.836, 0.793, and 0.840 on PDBbind-v2007, PDBbind-v2013, and PDBbind-v2016, respectively. The average *R* value across the three datasets is 0.823. The average *R* values of TopBP-ML^42^ and PPS-ML^22^ are 0.823 and 0.820, respectively, on these three datasets. Although PLLML is slightly inferior to PerSpect-ML on PDBbind-v2007, it yields significantly better predictions on the other two datasets: PDBbind-v2013 and PDBbind-v2016. The average *R* value of PLLML across the three datasets is 0.834, which is much higher than that of these models, as displayed in Table 2. These results highlight the effectiveness of PLLML as a novel analytical tool and demonstrate its potential for developing state-of-the-art models for binding affinity prediction.

**Figure 2:**
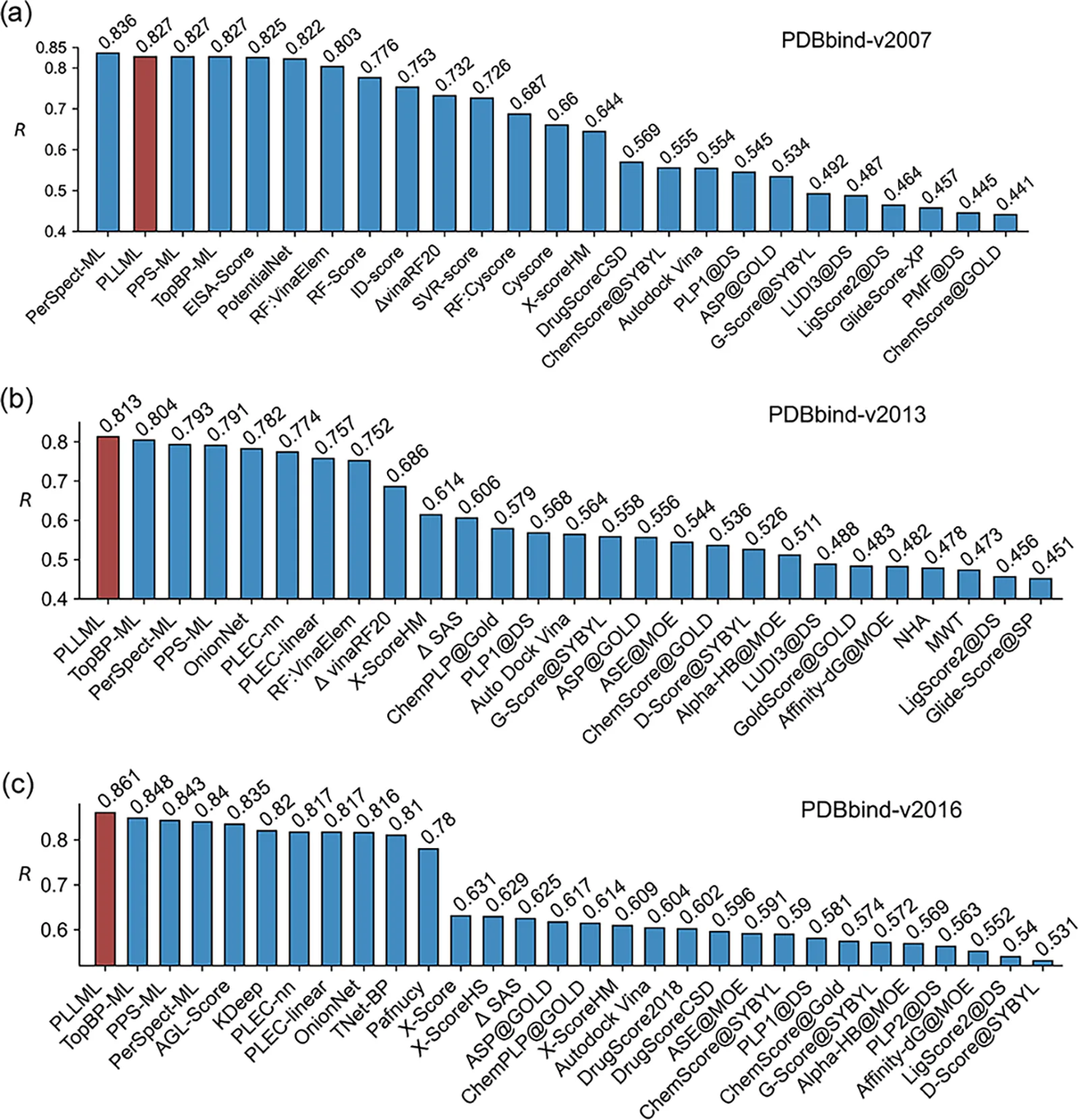
Comparison of the predictive performance of the PLLML model with other published models in terms of the Pearson correlation coefficient (*R*) on three PDBbind datasets.

**Table 2:** Comparison of Our PLLML Model (PLL + Transformer) with Advanced Mathematical Machine Learning Models^a^ ^21,22,42^

dataset	v2007	v2013	v2016	average
PLLML	0.827	**0.813**	**0.861**	**0.834**
PerSpect-ML^21^	**0.836**	0.793	0.840	0.823
TopBP-ML^42^	0.818	0.804	0.848	0.823
PPS-ML^22^	0.827	0.791	0.843	0.820

a The best-performing models are highlighted in bold.

## 3 Methods

In this section, we first present the information for the three PDBbind benchmark datasets. Then, we provide an overview of the persistent local Laplacian theory (PLL). Next, we describe the molecular featurization based on PLL, followed by natural language processing (NLP) molecular descriptors. Machine learning models are developed by combining these molecular descriptors with advanced machine learning algorithms (see Figure 3).

**Figure 3:**
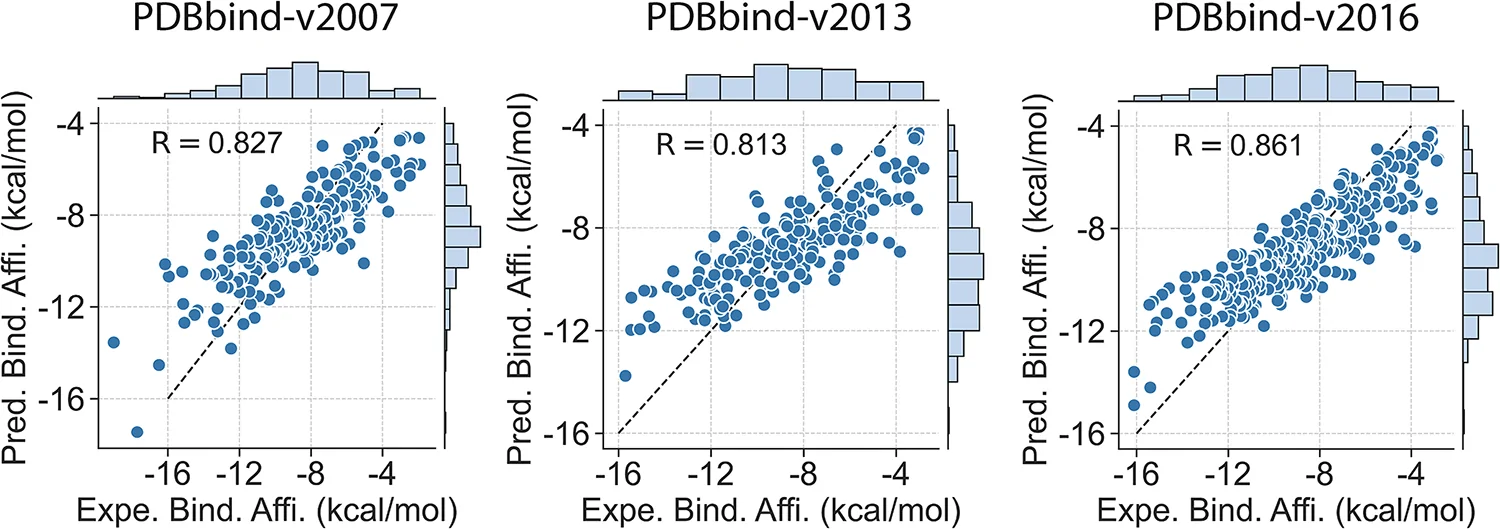
Comparison between the experimental and predicted binding affinities from our PLLML model on three PDBbind datasets.

### 3.1 Datasets

The PDBbind database^36^ is a widely recognized, curated resource that systematically collects experimentally determined 3D structures of protein–ligand complexes alongside their corresponding binding affinity data, e.g., dissociation constants *K*_d_, inhibition constants *K*_i_, and Gibbs free energy changes Δ*G*. We consider three widely used benchmark datasets, PDBbind-v2007, PDBbind-v2013, and PDBbind-v2016, to test the performance of our PLL method on protein–ligand binding affinity prediction. The dataset details are shown in Table 3.

**Table 3:** Details of the Datasets Utilized for Benchmark Tests in This Study

dataset	total	training set	test set
PDBbind-v2007 ^44^	1300	1105	195
PDBbind-v2013 ^45^	2959	2764	195
PDBbind-v2016 ^46^	4057	3767	290

### 3.2 Persistent Local Laplacian Theory

Persistent local Laplacian theory (PLL)^34^ characterizes both local topological structures and geometric information in datasets through the spectral analysis of local Laplacian matrices. This theory is one significant advancement of the two classes of prevalent TDA methods, including persistent homology^29,30^ and persistent Laplacians.^23,33^ Further details on the mathematical foundations and advantages of PLL are provided in the Supporting Information. Below, we present a brief overview of PLL; additional details can be found in Sections 2 and 3 of the Supporting Information.

First, the Laplacian on a simplicial complex can be viewed as a high-dimensional generalization of the graph Laplacian. For a simplicial complex K with the standard inner product defined on its chain complex $C_{*}\left(K\right)$ , the *Laplacian* on K is given by

$$\Delta_{k}=\partial_{k}^{*}\partial_{k}+\partial_{k+1}\partial_{k+1}^{*},k\geq 0$$

Here, ∂_*k*_ is the boundary map and (·)* denotes the adjoint operator with respect to the inner product.

Let L=K\{v} be the maximal subcomplex of K that does not contain the vertex *v*. Then the relative complex ${\left(C_{k}\left(K,L\right),{\mathrm{\partial ̅}}_{k}\right)}_{k\geq 0}$ associated with K relative to L constitutes a chain complex. Analogous to the construction of the simplicial Laplacian, we define the *local Laplacian at v* as a self-adjoint operator $\Delta_{k}^{K,v}:C_{k}\left(K,K\backslash \left\{v\right\}\right)\to C_{k}\left(K,K\backslash \left\{v\right\}\right)$ , which is expressed by

$$\Delta_{k}^{K,v}={\mathrm{\partial ̅}}_{k+1}{\mathrm{\partial ̅}}_{k+1}^{*}+{\mathrm{\partial ̅}}_{k}^{*}{\mathrm{\partial ̅}}_{k},k\geq 0$$

Now, consider a filtration of simplicial complexes

$$K_{0}\subseteq K_{1}\subseteq ...\subseteq K_{m}$$

For any vertex $v\in K_{0}$ , this yields a corresponding filtration of the relative chain complexes

$$C_{*}\left(K_{0},K_{0}\backslash \left\{v\right\}\right)\subseteq C_{*}\left(K_{1},K_{1}\backslash \left\{v\right\}\right)\subseteq ...\subseteq C_{*}\left(K_{m},K_{m}\backslash \left\{v\right\}\right)$$

Following the idea of the classical persistent Laplacian, for any indices *s* ≤ *t*, we can define a self-adjoint and positive semidefinite operator

$$\Delta_{k}^{s,t}\left(K_{•};v\right):C_{k}\left(K_{s},K_{s}\backslash \left\{v\right\}\right)\to C_{k}\left(K_{s},K_{s}\backslash \left\{v\right\}\right)$$

This operator is referred to as the persistent local Laplacian. This Laplacian encodes both the local information at the vertex *v* and the evolution of the persistence process. Its harmonic component is isomorphic to the persistent local homology, while its nonharmonic spectrum captures the local geometric features of the underlying structure (see the Supporting Information for theoretical details).

It is relatively difficult to understand and compute the PLL directly. Fortunately, the PLL can be evaluated using the persistent link Laplacian. For a simplicial complex K and a vertex v∈K , the link complex ${\mathrm{Lk}}_{K}\left(v\right)$ is defined as the subcomplex K consisting of all simplices *σ* such that *v* ∉ *σ* and σ∪{v}∈K . Accordingly, the above filtration of simplicial complexes gives rise to a filtration of link complexes

$${\mathrm{Lk}}_{K_{0}}\left(v\right)\to {\mathrm{Lk}}_{K_{1}}\left(v\right)\to ...\to {\mathrm{Lk}}_{K_{m}}\left(v\right)$$

Furthermore, we analogously define the persistent link Laplacian as a self-adjoint and positive semidefinite operator

$$\Delta_{k-1}^{s,t}\left({\mathrm{Lk}}_{K_{•}}\left(v\right)\right):C_{k-1}\left({\mathrm{Lk}}_{K_{s}}\left(v\right)\right)\to C_{k-1}\left({\mathrm{Lk}}_{K_{s}}\left(v\right)\right)$$

A key result establishes a unitary equivalence between the (*s*, *t*)-persistent local Laplacian and the (*s*, *t*)-persistent link Laplacian. Beyond its theoretical significance, the persistent link Laplacian provides a more efficient mathematical approach to computing persistent local spectral features. By virtue of the unitary equivalence established above, this formulation enables the development of more-efficient numerical algorithms. These algorithms, in turn, provide a solid mathematical foundation for large-scale data analysis and address the computational challenges arising from massive datasets in persistent topology applications.

### 3.3 Persistent Local Laplacian Molecular Descriptors

There are various types of intra- and intermolecular interactions, including hydrogen bonding, electrostatic forces, van der Waals forces, and hydrophobic/hydrophilic interactions. To effectively characterize these critical interactions, element-specific modeling was developed in our earlier work,^39^ where it demonstrated strong predictive power. In this study, we integrate element-specific modeling with the proposed persistent local Laplacian framework to capture and encode these interactions into molecular descriptors. We employ the persistent link Laplacian to compute the persistent local Laplacian, and the resulting eigenvalue information is used to characterize molecular structures. Specifically, we partition atoms in a protein–ligand complex into element-based groups. The four most common atom types in proteins are {C, N, O, S}, while ligands frequently contain {C, N, O, S, P, Cl, Br, I, H}. This yields 40 possible protein–ligand atom-type interaction pairs. A standard cutoff distance of 12 Å from the ligand is used to collect neighboring protein atoms. For each resulting 3D point cloud associated with an atom-type pair, we apply the persistent link Laplacian to generate a vectorized representation. The concatenation of the vectorized features from all 40 atom-type combinations forms the molecular descriptor for a protein–ligand complex.

Rather than with the persistent scales, we generate local Laplacian features of point clouds at each filtration parameter. Specifically, for each protein–ligand atom-type combination, we perform persistent link Laplacian analysis centered at every atom *v*, using a Vietoris–Rips complex with a filtration radius ranging from 2 to 6 Å in increments of 0.5 Å. For a given filtration radius, we obtain the eigenvalues of the dimension-1 local Laplacian matrix $\Delta_{1}^{s,s}\left(K_{•};v\right)$ (computed through link Laplacian $\Delta_{0}^{s,s}\left({\mathrm{Lk}}_{K_{•}}\left(v\right)\right)$ ) and summarize them using nine statistical descriptors, including the sum, mean, median, standard deviation, maximum, minimum, and variance, as well as the sum of squared positive eigenvalues and the number of zero eigenvalues. These nine values form the feature representation for each atom. To obtain a scalable representation for one atomic point cloud, we categorize atoms into two groups—protein atoms and ligand atoms. For each of the nine per-atom feature types, we compute two aggregate statistics (sum and mean) across all atoms in each group. This yields 18 features for protein atoms and 18 features for ligand atoms, giving a total of 36 features at a single filtration radius. Because we use nine filtration radii, we obtain 324 features per point-cloud combination. In total, 12,960 features are generated to characterize a protein–ligand complex. We also construct ligand-specific molecular descriptors using PLL analysis. For each ligand, we consider the point cloud formed by atoms of types {C, N, O, S, P, Cl, Br, H, F, I}, and apply a Vietoris–Rips filtration from 2 to 6 Å with a step size of 0.5 Å. We further perform element-specific characterization using 13 atom subsets: {C}, {N}, {O}, {S}, {C, N}, {C, O}, {C, S}, {N, O}, {N, S}, {O, S}, {N, P}, {F, Cl, Br, I}, and {C, O, N, S, F, P, Cl, Br, I}. For each subset, we obtain the same nine statistical descriptors of the dimension-1 local Laplacian $\Delta_{1}^{s,s}\left(K_{•};v\right)$ eigenvalues at each filtration radius, and then take the sum and mean of each descriptor across all atoms in the subset. This procedure yields 2106 features for each ligand. Finally, we concatenate the 12,960 protein–ligand complex features with the 2106 ligand features, producing a representation that captures both intermolecular and intramolecular interactions for each protein–ligand pair.

In Figure 4, we illustrate the characterization of the C*α* atoms in protein 6L9D using the persistent link Laplacian. Under a filtration process with radius up to seven, the eigenvalues of the 0-dimensional link Laplacian $\Delta_{0}^{s,s}\left({\mathrm{Lk}}_{K_{•}}\left(v\right)\right)$ (*L*_0_) and the 1-dimensional link Laplacian $\Delta_{1}^{s,s}\left({\mathrm{Lk}}_{K_{•}}\left(v\right)\right)$ (*L*_1_) exhibit distinct patterns, particularly when the filtration radius exceeds 4. However, in this work, we use only the eigenvalue information from $\Delta_{0}^{s,s}\left({\mathrm{Lk}}_{K_{•}}\left(v\right)\right)$ to reduce computational cost and mitigate overfitting.

**Figure 4:**
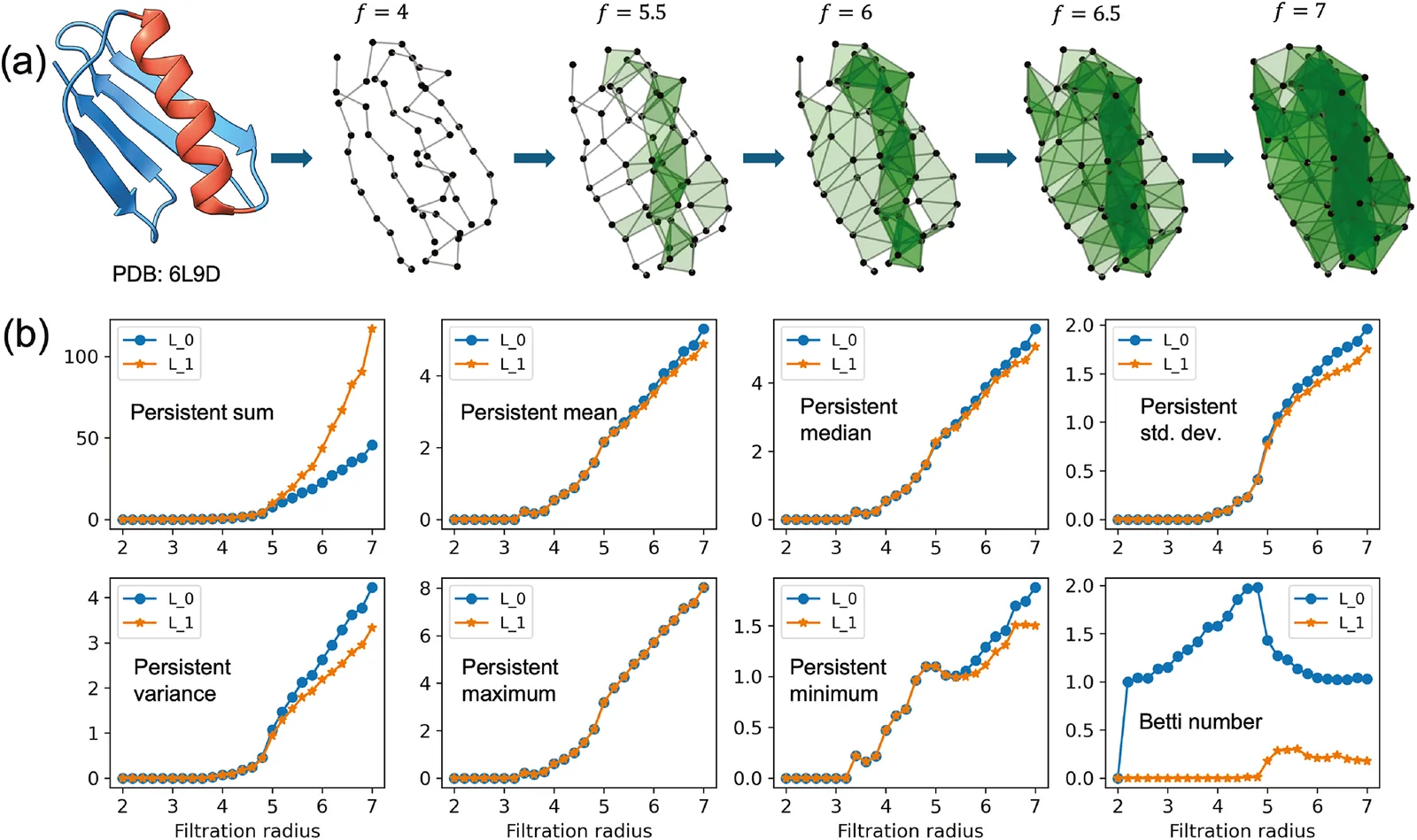
Illustration of the mean persistent quantities—including sum, mean, median, standard deviation, variance, maximum, minimum, and Betti numbers—derived from eigenvalues of persistent link Laplacians in modeling Cα atoms in protein 6L9D. (a) The filtration of Cα atoms in protein 6L9D. (b) The evolution of these persistent quantities along the filtration process. *L*_0_ and *L*_1_ curves represent the statistical summaries of eigenvalue of 0-dimensional and 1-dimensional link Laplacian matrices.

### 3.4 Natural Language Processing (NLP) Molecular Descriptors

Transformer models, a core class of natural language processing (NLP) techniques, have recently emerged as powerful tools in molecular biosciences. In this work, we employ pretrained transformer models to extract sequence-based features for both proteins^40^ and ligands.^41^ The inputs to these models are amino acid sequences for proteins and SMILES strings for ligands. By concatenating the molecular descriptors generated from the pretrained models (ESM)^40^ for protein sequences and ligand SMILES strings,^41^ we obtain a unified sequence-based representation of each protein–ligand complex.

### 3.5 Machine Learning Modeling

To construct our predictive models, we adopt the gradient boosting decision tree (GBDT) framework, implemented through the scikit-learn library (v1.5.1). GBDT offers strong performance in a wide range of regression tasks due to its resilience to overfitting, minimal sensitivity to hyperparameter tuning, and a straightforward training procedure. The method iteratively builds an ensemble of decision trees where each tree is trained to correct the residual errors of the previous ones. Although individual trees act as weak learners, their sequential combination forms a powerful model capable of capturing complex, nonlinear relationships. Both the PLL-derived structural features and transformer-generated sequence features are independently used as inputs to train GBDT regressors. The specific hyperparameter settings employed in our experiments are summarized in Table 4. The formulations of the two evaluation indicators, including PCC and RMSE, are given in Section 6 in the Supporting Information.

**Table 4:** Hyperparameters Used for Building Gradient Boosting Regression Models^a^

no. of estimators	max depth	min. sample split	learning rate
30,000/20,000	7	5	0.002
max features	dubsample size	repetition	
square root	0.8	10 times	

a Tree numbers are set to be 30,000 and 20,000, respectively, for PLL and transformer-based biomolecular modeling.

## 4 Discussion

### 4.1 PLL Modeling Interpretability

The PLL framework offers excellent interpretability for modeling molecular structures, which motivates its application to molecular descriptor design. To illustrate the PLL modeling process, we consider a point-cloud example shown in Figure 5. Figure 5a shows 4 types of building blocks in a simplicial complex, while panel b illustrates a filtration process using Vietoris–Rips complexes. In Figure 5c, a Vietoris–Rips filtration is applied to a point cloud consisting of 12 points, where six points lie on a circle of radius 1 and the remaining six lie on a circle of radius 0.5. Link complex ${\mathrm{Lk}}_{K_{•}}\left(v\right)$ encodes the local topological and geometric structures in the neighborhood of a point of interest (*v*, highlighted in red). It is composed of edges and triangles around *v*. As the filtration radius increases, link complexes of progressively larger sizes are formed around *v*.

**Figure 5:**
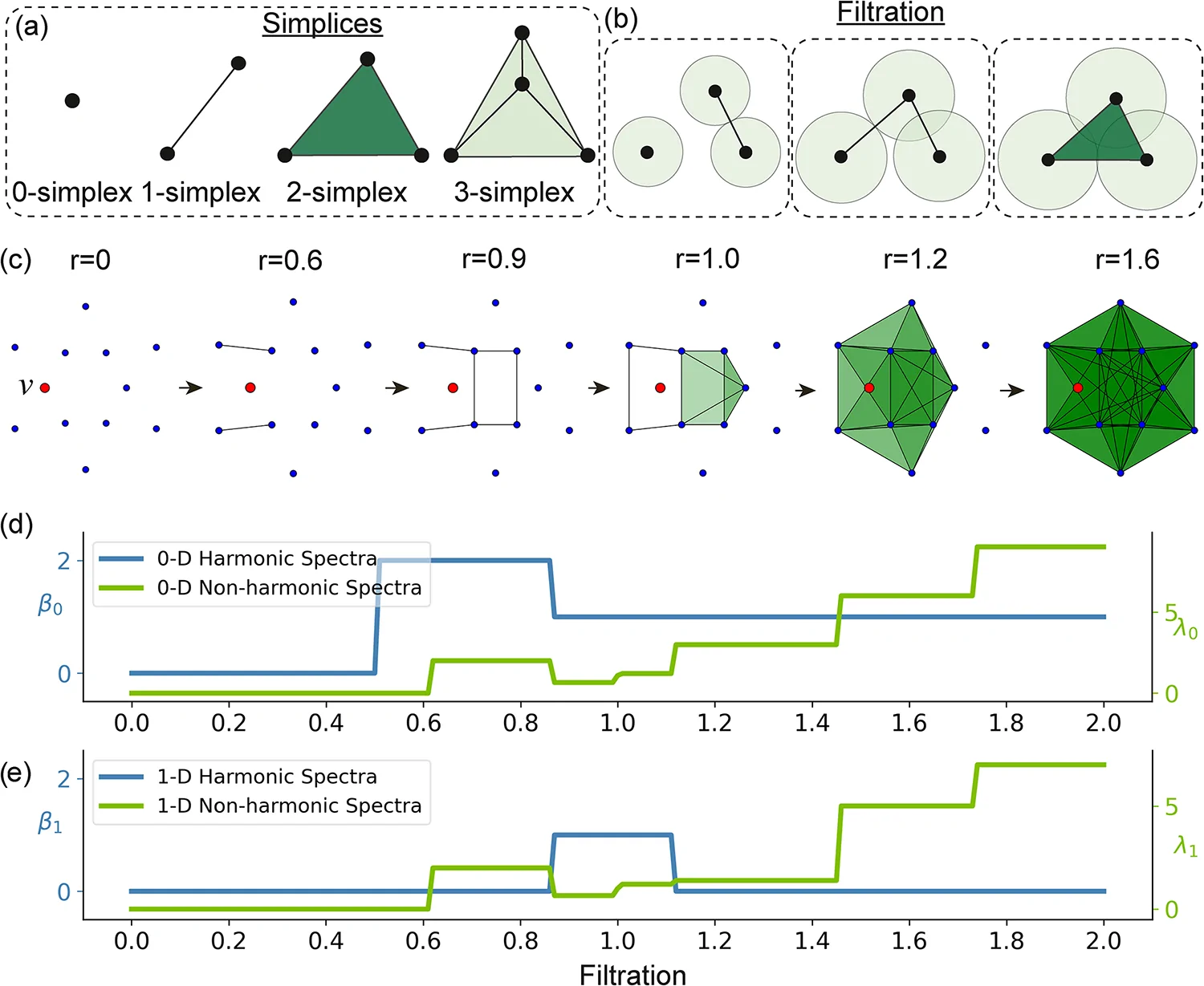
Illustration of persistent local Laplacians (PLL) at one point in a point cloud. (a) 4 types of building blocks in a simplicial complex. (b) Filtration process of the Vietoris–Rips complex constructed from a point cloud of 12 points. (c) Among the 12 points, six lie on a circle of radius 1 and the other six lie on a circle of radius 0.5. The points on the smaller circle are obtained by a rotation of $\frac{\pi }{6}$ so that each point on the smaller circle lies on the same radial line from the origin as a corresponding point on the larger circle. (d) Analysis of zero-dimensional spectral information of local Laplacian matrices defined for the red point *v* over the filtration process. (e) Analysis of one-dimensional persistent Laplacian spectra of PLL defined for point *v* over the filtration process.

For each link complex, local Laplacian matrices of different dimensions can be constructed, and their spectral properties quantitatively characterize the local structural features surrounding v. The Betti number β_0_, defined as the number of zero eigenvalues of the link Laplacian matrix $\Delta_{0}^{s,s}\left({\mathrm{Lk}}_{K_{•}}\left(v\right)\right)$ , indicates the number of independent connected components in the link complex. We plot the variation of β_0_ along the filtration as the blue curve in Figure 5d. At filtration radius *r* = 0.0, the link complex of the red point is empty, yielding β_0_ = 0 in Figure 5d. When the filtration radius increases to *r* = 0.6, two independent edges appear in the link complex, resulting in β_0_ = 2. As the filtration radius further increases to *r* = 1.0, these edges become connected, forming a single connected component and reducing β_0_ to 1. Beyond the zero eigenvalues, the nonzero eigenvalues of local Laplacian matrices provide additional geometric information and reveal the homotopic evolution of the link sets throughout the filtration. In particular, the smallest nonzero eigenvalues Λ_0_ reflect geometric changes in the local structures as the filtration progresses, as shown by the green curve in Figure 5d. The values of Λ_0_ capture connectivity changes in these link complexes.

Similarly, the Betti number β_1_, defined as the number of zero eigenvalues of the link Laplacian matrix $\Delta_{1}^{s,s}\left({\mathrm{Lk}}_{K_{•}}\left(v\right)\right)$ , quantifies the number of independent loops in the link complex. We compute these values along the filtration, as shown by the blue curve in Figure 5e. At a filtration radius of *r* = 1.0, a single loop appears in the link complex (see the third figure in Figure 5c), giving *β*_1_ = 1. These spectral quantities derived from the link Laplacians $\Delta_{k}^{s,s}\left({\mathrm{Lk}}_{K_{•}}\left(v\right)\right)$ provide both topological and geometric insights into local structures, making them well-suited for biomolecular structural modeling.

### 4.2 Filtration Impacts on Modeling Performance

The performance of persistent local Laplacians (PLLs) for protein–ligand binding affinity prediction depends on the parameterization of the filtration. To identify an effective filtration range, we evaluated model performance at different filtration radii on three PDBbind benchmark datasets. We considered two types of models: one using molecular features derived solely from the protein–ligand complex, and another incorporating additional ligand-specific molecular features. As shown in Figure 6, the predictive performance of both PLL-based models improves as the filtration radius increases from 4 to 6 Å. However, performance declines slightly beyond 6 Å on PDBbind-v2007 and PDBbind-v2016, whereas on PDBbind-v2013 the model achieves its best performance at 7 Å, with only marginal improvement over 6 Å. These results suggest that a filtration radius of 6Å is optimal for PLL-based modeling of protein–ligand binding. Using an upper filtration radius of 6Å also enables efficient feature construction while maintaining the ability to capture essential molecular interactions.

**Figure 6:**
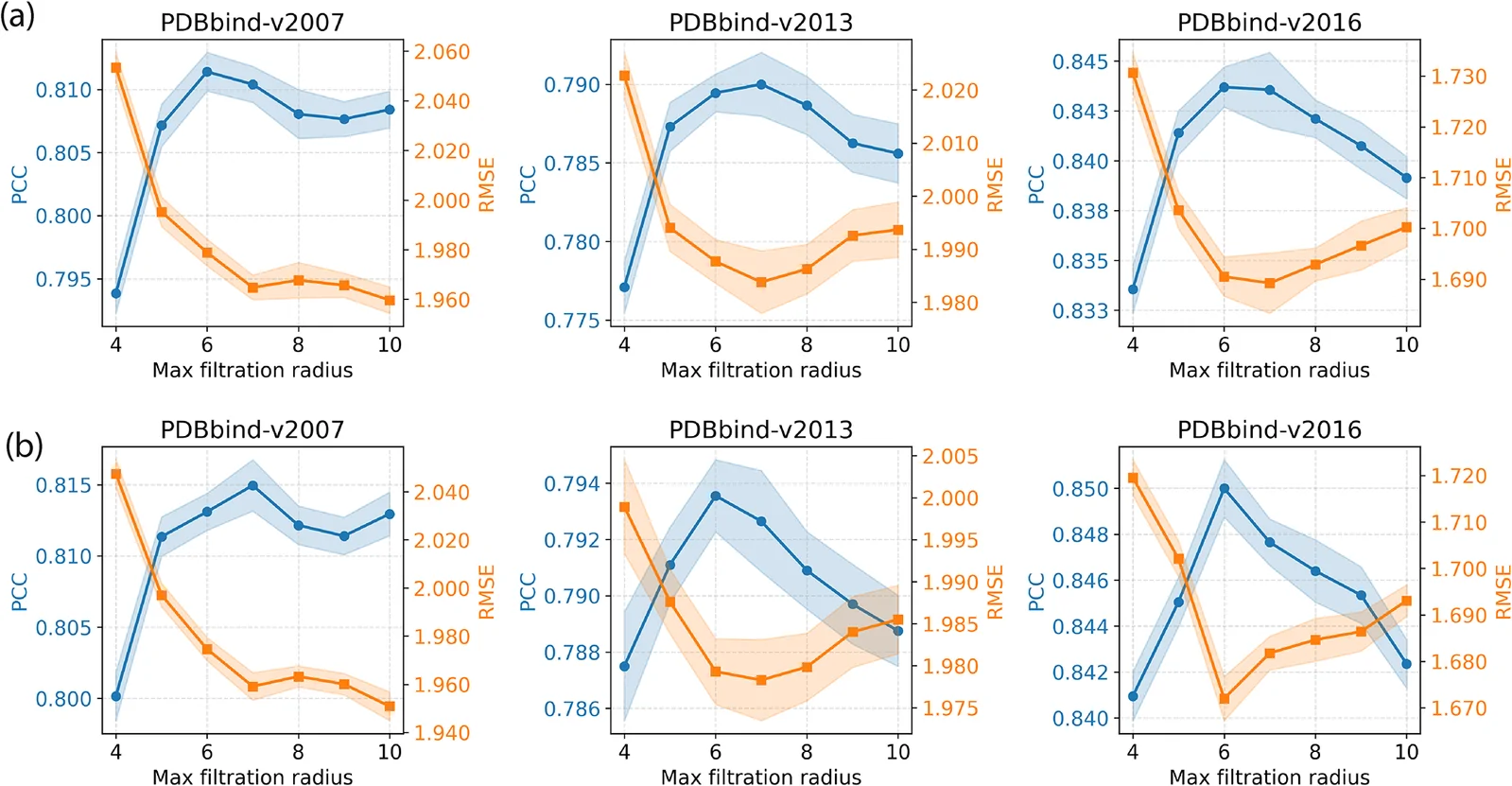
Effect of varying the upper bound of the filtration radius on the predictive performance of the persistent local Laplacian models across three PDBbind datasets. (a) The performance of PLL models using the molecular features solely based on the characterization of protein–ligand complex without additional features of the ligand molecule. (b) The performance of PLL models using the molecular features based on the characterization of protein–ligand complex without additional features of the ligand molecule. The dotted values on the curves represent the average Pearson correlation coefficient (PCC) obtained from 20 models with different random seeds. The shaded width of each curve indicates the standard deviation of these 20 PCC values. The detailed numerical data is provided in Tables S3 and S4.

### 4.3 Computational Complexity Analysis

The computational complexity of the proposed persistent link Laplacian is significantly lower than that of the standard persistent Laplacian method.^21,23^ In both approaches, the computational cost primarily arises from two sources: (i) construction of the Laplacian matrix and (ii) computation of its full spectrum of eigenvalues. The persistent link Laplacian exhibits a lower complexity in both aspects.

The key advantage of local Laplacians becomes evident when considering large-scale systems. The global spectral complexity of the persistent Laplacian is *O*(*n*_*i*_^3^), where *n*_*i*_ denotes the number of *i*-simplices in the simplicial complex. In contrast, the spectral complexity of the link Laplacian is much lower. Suppose *q* is a small constant representing the maximum number of simplices incident to a single vertex in the simplicial complex. Then we typically have *n*_0_, *n*_1_, *n*_2_ ≫ *q*. In this setting, the spectral complexity for *n*_0_ link Laplacian matrices, which is *O*(*n*_0_ *q* ^3^), is significantly smaller than the global complexity *O*(*n*_0_^3^) or *O*(*n*_1_^3^), while the ratio *n*_0_/*n*_1_ scales with the size of the entire complex. This substantial reduction in computational complexity highlights the practicality of local Laplacians for large-scale topological analysis. Detailed analyses and comparisons of the computational complexity for both methods are provided in Section 3.3 and Table S1. We also made some efficiency comparisons between the persistent local Laplacian and the classical persistent Laplacian method via ten protein–ligand complexes. The CPU time comparison of the two methods, as shown in Table S5, demonstrates the efficiency of our proposed persistent local Laplacian method.

## Supplementary Material



## Data Availability

All data and the code needed to reproduce this paper’s result can be found at https://github.com/hfeng2uncc/PLLML.git. The PDBbind datasets are available at https://www.pdbbind-plus.org.cn/.

## Notes

Story of local topological spectral analysis; quick overview from homology to persistent Laplacian; additional information on local spectral analysis; result analysis of PLL on protein–ligand binding affinity; molecular large language models; evaluation metrics; relationship between a series of Laplacian methods and homology methods (Figure S1); and illustration of relative homology, local homology, and the link (Figure S2). Computational complexity of Laplacian matrix and spectral calculation (Table S1); modeling performance of different strategies on the test sets of the three PDBbind benchmark datasets (Table S2); effect of filtration radius on PLL predictive performance (Tables S3 and S4); and CPU time comparisons between persistent local Laplacian (PLL) and persistent Laplacian (PL) across several large proteins (Table S5) (PDF)
