# Supplementary material for: Persistent Local Laplacian Prediction of Protein–Ligand Binding Affinities
Source: J Chem Inf Model. 2026 Sep 4;66(18):11767–77. doi: 10.1021/acs.jcim.6c02460 (PMC13625961; doi:10.1021/acs.jcim.6c02460)
Supplement: Supplementary file 1 [file ci6c02460_si_001.pdf]

Supporting Information

Persistent local Laplacian prediction of protein-ligand binding affinities

Jian Liu[1] and Hongsong Feng[2] *,
[1] Mathematical Science Research Center,
Chongqing University of Technology, Chongqing 400054, P. R. China
[2] Department of Mathematics and Statistics,
University of North Carolina at Charlotte, Charlotte, NC 28223, USA

August 21, 2026

*Corresponding author: Hongsong Feng (hfeng2@charlotte.edu).

Contents

1 The story of local topological spectral analysis **2**

2 A quick overview : from homology to persistent Laplacian **2**

2.1 Homology and persistent homology . . . 2

2.1.1 Simplicial complex . . . 3

2.1.2 Simplicial homology . . . 4

2.1.3 Persistent homology . . . 4

2.2 Persistent Laplacian . . . 5

2.2.1 Simplicial Laplacian . . . 5

2.2.2 Persistent Laplacian . . . 5

2.2.3 Persistent Hodge isomorphism . . . 6

3 Additional information on local spectral analysis **6**

3.1 Local topological invariant . . . 6

3.1.1 Relative homology . . . 6

3.1.2 Local homology . . . 7

3.1.3 Local Laplacian . . . 7

3.1.4 Link complex . . . 8

3.1.5 Connection to link Laplacian . . . 8

3.2 Persistent local spectral analysis . . . 9

3.2.1 Persistent Local Laplacian . . . 9

3.2.2 Persistent Link Laplacian . . . 11

3.3 Computational complexity analysis . . . 11

3.3.1 Computational complexity of Laplacian matrix . . . 12

3.3.2 Spectra calculation complexity of Laplacian matrices . . . 13

4 Result analysis of PLL on protein-ligand binding affinity **14**

4.1 Comparisons of different model strategies. . . . 14

4.2 Impacts of filtration ranges on PLL models. . . . 14

4.3 Computational efficiency comparisons between PLL and persistent Laplacian . . . 14

5 Molecular large language models **16**

5.1 Transformer-based protein language model . . . 16

5.2 Transformer-based small molecular language model . . . 17

6 Evaluation metrics **17**

1 The story of local topological spectral analysis

Homology is one of the most widely used topological invariants for characterizing spatial structures, encoding information about connected components, loops, and cavities within a space. Topological features reflect the intrinsic and holistic properties of data at the structural level, exhibiting strong stability against perturbations. However, traditional homology is overly coarse-grained and thus difficult to apply directly to data analysis tasks. In contrast, persistent homology, first introduced by Carlsson, Edelsbrunner, and colleagues [1, 2], characterizes data through multi-scale topological features. Essentially, the multi-scale nature of persistent homology inherently captures partial geometric characteristics of the data, which has led to its remarkable success in data science, articularly in the analysis of complex structures, and rapid development in recent decades.

Subsequently, persistent Laplacian has also seen significant advancements in topological data analysis (TDA) [3, 4, 5]. The core reason for its success lies in the fact that the Laplacian operator encodes more geometric information than traditional homology. Notably, a natural isomorphism exists between the harmonic space of the Laplacian and homology groups: in other words, the harmonic component of the persistent Laplacian retains all information from persistent homology, while its non-harmonic component captures the geometric properties of the data.

Currently, persistent homology and persistent Laplacian are the most prevalent tools in TDA. Nevertheless, their computational complexity poses significant limitations when applied to large-scale systems. Furthermore, in large systems, the mutual influence between distinct local regions diminishes as the distance between them increases, while the demand for localized information becomes increasingly critical. To address these challenges, we propose the Persistent Local Laplacian (PLL) [6], a topology-spectrum method tailored to local structures for capturing the local geometry and intermolecular interactions of molecular systems. The core idea of PLL is to extract localized information from data by leveraging local topological invariants and geometric features. This approach offers four key advantages:

(a) It mitigates the insensitivity of traditional persistent homology and persistent Laplacian to the local structural information of data;

(b) The small size of local subsystems leads to substantial improvements in the efficiency and computational complexity of local feature extraction;

(c) Local feature calculations for different vertices/points can be executed in parallel, further accelerating computations;

(d) Local features exhibit excellent scalability—extensions of the system do not alter precomputed local features, eliminating the need for redundant recalculation.

Figure S1 illustrates how the PLL integrates topological and geometric information, while also incorporating persistence and locality into data analysis. This represents a theoretical advancement and entails substantial intrinsic benefits for practical applications: it enables the extraction of localized features with enhanced efficiency, supports parallel computation, and exhibits strong scalability.

2 A quick overview : from homology to persistent Laplacian

2.1 Homology and persistent homology

Let $\mathbb{F}$ be the base field, typically $\mathbb{Z}/2\mathbb{Z}$ or $\mathbb{R}$.

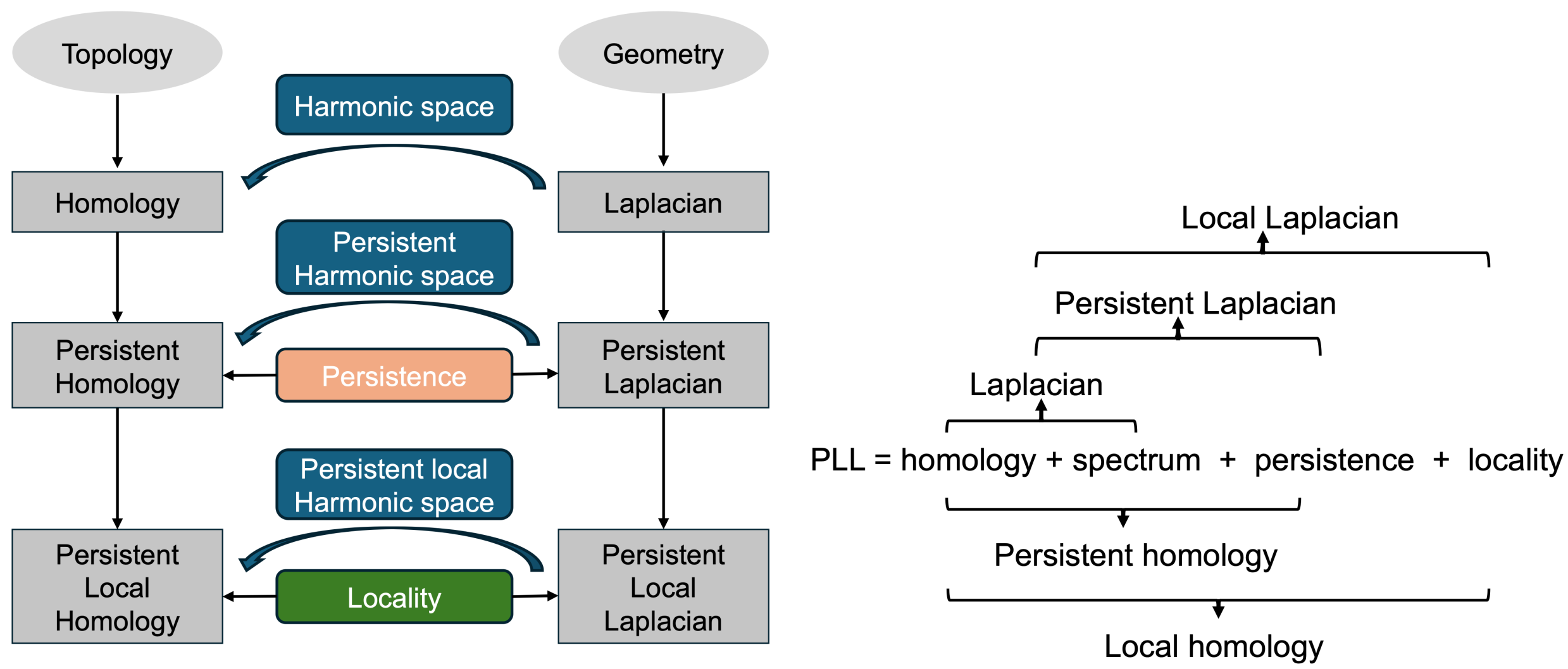

Figure S1: Relationship between persistent local Laplacian and other topological data analysis methods. The left figure shows how homology theories are related to Laplacian theories in horizontal view. Vertically, we see how homology/Laplacian, persistent homology/Laplacian, and persistent local homology/Laplacian evolve. The right figure shows how persistent local Laplacian integrates elements from different theories.

2.1.1 Simplicial complex

Let $d \in \mathbb{N}$ and $\{v_0, v_1, \ldots, v_n\}$ be a geometrically independent point set in the Euclidean space $\mathbb{R}^d$. The convex hull

$$\sigma = \left\{ \sum_{i=0}^{n} \lambda_i v_i \mid \lambda_i \geq 0, \sum_{i=0}^{n} \lambda_i = 1 \right\}$$

is called an n-dimensional simplex (denoted $[v_0, v_1, \ldots, v_n]$). In the usual sense, a single point is a 0-simplex, a line segment is a 1-simplex, a triangle (filled) is a 2-simplex, a tetrahedron is a 3-simplex, and so forth. A simplex τ is a *face* of σ if the vertex set of τ is a subset of that of σ; a *proper face* is a face with $\dim(\tau) < \dim(\sigma)$.

A collection $\mathcal{K}$ of simplices in $\mathbb{R}^d$ is defined as a *simplicial complex* if and only if it satisfies two axioms:

(1) If $\sigma \in \mathcal{K}$, then all faces of σ are also in $\mathcal{K}$;

(2) For any $\sigma_1, \sigma_2 \in \mathcal{K}$, their intersection $\sigma_1 \cap \sigma_2$ is either empty or a common face of both simplices.

Example 2.1 (Vietoris-Rips Complex)**.** Let $X = \{x_1, x_2, \ldots, x_m\}$ be a finite point set in a metric space (X, d). For a fixed parameter $\epsilon > 0$, the Vietoris-Rips complex $\mathrm{VR}(X, \epsilon)$ is constructed as follows:

- All single points in X are 0-simplices of $\mathrm{VR}(X, \epsilon)$;
- A subset $\{x_{i_0}, x_{i_1}, \ldots, x_{i_k}\} \subseteq X$ forms a k-simplex if and only if the pairwise distance $d(x_{i_p}, x_{i_q}) \leq \epsilon$ for all $0 \leq p, q \leq k$.

Simplicial complexes are the most fundamental topological model for characterizing data in topological data analysis. Compared with graphs (which only capture pairwise relationships via

edges, i.e., 1-simplices), simplicial complexes can explicitly represent high-dimensional structures of data (e.g., 2-simplices for triangular correlations, 3-simplices for tetrahedral correlations). Among various simplicial complex constructions, the Vietoris-Rips complex is the most widely used.

2.1.2 Simplicial homology

For a simplicial complex $\mathcal{K}$, let $C_k(\mathcal{K};\mathbb{F})$ denote the k-th *simplicial chain group* (or chain complex) over a field $\mathbb{F}$, which is the vector space spanned by all k-simplices in $\mathcal{K}$. The *boundary operator* $\partial_k : C_k(\mathcal{K};\mathbb{F}) \to C_{k-1}(\mathcal{K};\mathbb{F})$ is the linear map

$$\partial_k[v_0, v_1, \ldots, v_k] = \sum_{i=0}^{k}(-1)^i[v_0, \ldots, \widehat{v_i}, \ldots, v_k],$$

where $\widehat{v_i}$ denotes the omission of vertex v_i. One can verify that $\partial_{k-1} \circ \partial_k = 0$, meaning the boundary of a boundary is empty.

The k-th *simplicial homology group* $H_k(\mathcal{K};\mathbb{F})$ is defined as the quotient space

$$H_k(\mathcal{K};\mathbb{F}) = \frac{\ker \partial_k}{\text{im } \partial_{k+1}},$$

where $\ker \partial_k = \{x \in C_k(\mathcal{K};\mathbb{F}) \mid \partial_k x = 0\}$ and $\text{im}\, \partial_{k+1} = \{\partial_{k+1} x \mid x \in C_{k+1}(\mathcal{K};\mathbb{F})\}$ are k-chains that are boundaries of $(k+1)$-chains. The *rank* of $H_k(\mathcal{K};\mathbb{F})$, denoted $\beta_k(\mathcal{K})$, is called the k-th Betti number. Precisely, $\beta_0(\mathcal{K})$ counts the number of connected components of $\mathcal{K}$, $\beta_1(\mathcal{K})$ the number of 1-dimensional holes in $\mathcal{K}$, and $\beta_2(\mathcal{K})$ the number of 2-dimensional voids in $\mathcal{K}$.

2.1.3 Persistent homology

Classical homology is a coarse topological invariant, whereas persistent homology introduces the concept of persistence to characterize far more nuanced topological features of data. Consider a filtration of simplicial complexes $\mathcal{K}_\bullet = \{\mathcal{K}_t\}_{t=0}^{m}$.

For each integer $k \geq 0$ and indices $s \leq t$, the inclusion map $\mathcal{K}_s \hookrightarrow \mathcal{K}_t$ induces a homomorphism $f_{k;s,t} : H_k(\mathcal{K}_s;\mathbb{F}) \to H_k(\mathcal{K}_t;\mathbb{F})$. The (s,t)-*persistent homology* is defined as the image of this homomorphism

$$H_k^{s,t}(\mathcal{K}_\bullet;\mathbb{F}) := \text{im}\left(f_{k;s,t} : H_k(\mathcal{K}_s;\mathbb{F}) \to H_k(\mathcal{K}_t;\mathbb{F})\right), \quad k \geq 0.$$

For a homology generator ω, the smallest scale b at which ω first appears is referred to as its birth time, and the smallest scale d at which ω vanishes is called its death time. This birth-death information encodes how the topological features captured by persistent homology evolve across different scales. Notably, the death time d may be infinite ($d = \infty$), indicating that the generator never vanishes.

All birth-death pairs (b, d) of generators in persistent homology form the persistence diagram (PD), which encapsulates the scale-dependent topological characteristics of the data. Features with large persistence $(d - b)$ are deemed topologically meaningful (robust to noise), while those with small persistence are attributed to spurious noise. If each birth-death pair (b, d) is represented as a horizontal bar spanning from b to d on the real line, the collection of such bars is called a persistence barcode, which is regarded as the topological fingerprint of the persistent homology of the underlying data.

2.2 Persistent Laplacian

The persistent Laplacian extends the classical simplicial Laplacian to the technique of persistent homology, bridging spectral topology and persistent topological feature analysis.

2.2.1 Simplicial Laplacian

We set $\mathbb{F} = \mathbb{R}$. On the k-chain group $C_k(\mathcal{K};\mathbb{R})$, which is a real vector space spanned by the k-simplices of $\mathcal{K}$, we equip the *standard inner product*

$$\langle \sigma, \tau \rangle = \begin{cases} 1 & \text{if } \sigma = \tau, \\ 0 & \text{otherwise,} \end{cases}$$

for any k-simplices σ, τ in $\mathcal{K}$. With respect to this inner product, the adjoint operator $\partial_k^*\colon C_{k-1}(\mathcal{K};\mathbb{R}) \to C_k(\mathcal{K};\mathbb{R})$ of $\partial_k\colon C_k(\mathcal{K};\mathbb{R}) \to C_{k-1}(\mathcal{K};\mathbb{R})$ is defined as

$$\langle \partial_k x, y \rangle = \langle x, \partial_k^* y \rangle, \quad \forall x \in C_k(\mathcal{K};\mathbb{R}), y \in C_{k-1}(\mathcal{K};\mathbb{R}).$$

We then have the following adjoint sequence

$$C_{k+1}(\mathcal{K};\mathbb{R}) \underset{(\partial_{k+1})^*}{\overset{\partial_{k+1}}{\rightleftarrows}} C_k(\mathcal{K};\mathbb{R}) \underset{(\partial_k)^*}{\overset{\partial_k}{\rightleftarrows}} C_{k-1}(\mathcal{K};\mathbb{R}).$$

The k-th *simplicial Laplacian* (or combinatorial Laplacian) is given by

$$\Delta_k := \partial_k^* \partial_k + \partial_{k+1} \partial_{k+1}^*, \quad k \geq 0.$$

In particular, when $\mathcal{K}$ is a 1-dimensional simplicial complex, i.e., an undirected graph, its simplicial Laplacian coincides with the standard graph Laplacian.

2.2.2 Persistent Laplacian

Consider a filtration of simplicial complexes $\mathcal{K}_\bullet = \{\mathcal{K}_t\}_{t=0}^m$. For any $t \in \mathbb{R}$, let $C_k(\mathcal{K}_t;\mathbb{R})$ be the chain complex of $\mathcal{K}_t$. For any $s \leq t$, the inclusion $\mathcal{K}_s \hookrightarrow \mathcal{K}_t$ induces a natural chain map between the corresponding chain complexes

$$j_* : C_*(\mathcal{K}_s;\mathbb{R}) \hookrightarrow C_*(\mathcal{K}_t;\mathbb{R}).$$

By equipping the chain complexes with a standard inner product, $C_*(\mathcal{K}_s;\mathbb{R})$ can be regarded as an inner product subspace of $C_*(\mathcal{K}_t;\mathbb{R})$. We set

$$C_k^{s,t}(\mathcal{K}_\bullet;\mathbb{R}) = \{x \in C_k^t(\mathcal{K}_t;\mathbb{R}) \mid \partial_k^t x \in C_{k-1}^s(\mathcal{K}_s;\mathbb{R})\}.$$

Let $\partial_k^{s,t} : C_k^{s,t}(\mathcal{K}_\bullet;\mathbb{R}) \to C_{k-1}^s(\mathcal{K}_s;\mathbb{R})$ denote the restriction of the boundary operator ∂_k^t to the subspace $C_k^{s,t}(\mathcal{K}_\bullet;\mathbb{R})$. These spaces and operators are organized into the following commutative diagram:

$$\begin{array}{ccccccc}
C_{k+1}(\mathcal{K}_s;\mathbb{R}) & \xrightarrow{\partial_{k+1}^s} & C_k(\mathcal{K}_s;\mathbb{R}) & \xrightarrow{\partial_k^s} & C_{k-1}(\mathcal{K}_s;\mathbb{R}) & & \boxed{\Delta_k^s} \\
\downarrow & & \| & & \| & & \\
C_{k+1}^{s,t}(\mathcal{K}_\bullet;\mathbb{R}) & \xrightarrow{\partial_{k+1}^{s,t}} & C_k(\mathcal{K}_s;\mathbb{R}) & \xrightarrow{\partial_k^s} & C_{k-1}(\mathcal{K}_s;\mathbb{R}) & & \boxed{\Delta_k^{s,t}} \\
\downarrow & & \downarrow & & \downarrow & & \\
C_{k+1}(\mathcal{K}_t;\mathbb{R}) & \xrightarrow{\partial_{k+1}^t} & C_k(\mathcal{K}_t;\mathbb{R}) & \xrightarrow{\partial_k^t} & C_{k-1}(\mathcal{K}_t;\mathbb{R}) & & \boxed{\Delta_k^t}
\end{array}$$

In this diagram, the first and third rows lead to the standard simplicial Laplacians at filtration steps s and t, respectively. The second row defines the *persistent (simplicial) Laplacian*

$$\Delta_k^{s,t} := (\partial_k^s)^* \partial_k^s + \partial_{k+1}^{s,t} (\partial_{k+1}^{s,t})^*, \quad k \geq 0,$$

where $(\cdot)^*$ denotes the adjoint operator with respect to the inner product.

2.2.3 Persistent Hodge isomorphism

The persistent Laplacian $\Delta_k^{s,t} : C_k(\mathcal{K}_s;\mathbb{R}) \to C_k(\mathcal{K}_s;\mathbb{R})$ is a self-adjoint, semi-positive definite operator. This property implies that all its eigenvalues are non-negative. The set of all eigenvalues is referred to as the *persistent spectrum*. In particular, the eigenspace corresponding to the zero eigenvalue is known as the *persistent harmonic space*.

The persistent Hodge isomorphism theorem establishes a natural isomorphism between the persistent harmonic space and the persistent homology. This fundamental result ensures that all topological information from persistent homology can be recovered through the persistent Laplacian. Specifically:

Theorem 2.2 ([7]). *For any real numbers $s \leq t$, there exists a natural isomorphism*

$$\mathcal{H}_k^{s,t}(\mathcal{K}_\bullet;\mathbb{R}) \cong H_k^{s,t}(\mathcal{K}_\bullet;\mathbb{R})$$

between the persistent harmonic space and the persistent homology, where $\mathcal{H}_k^{s,t}(\mathcal{K}_\bullet;\mathbb{R}) := \ker \Delta_k^{s,t}$.

While the zero eigenvalues identify the persistent Betti numbers, the non-zero spectrum characterizes the underlying geometric information of the data, such as the connectivity strength between points and the geometric tightness of loops.

Compared to traditional persistent homology, the persistent Laplacian adds a spectral dimension to topological data analysis: it not only identifies the birth, death, and persistence of topological features but also provides a refined characterization of their geometric properties through its spectral distribution.

3 Additional information on local spectral analysis

3.1 Local topological invariant

3.1.1 Relative homology

Let $\mathcal{K}$ be a simplicial complex and $\mathcal{L} \subseteq \mathcal{K}$ a subcomplex. The chain complex $C_*(\mathcal{L};\mathbb{R})$ is a subcomplex of $C_*(\mathcal{K};\mathbb{R})$. For brevity, we write $C_*(\mathcal{K})$ for $C_*(\mathcal{K};\mathbb{R})$ (and $C_*(\mathcal{L})$ for $C_*(\mathcal{L};\mathbb{R})$) from now on. The *relative chain complex* is defined as the quotient space

$$C_k(\mathcal{K},\mathcal{L}) := C_k(\mathcal{K})/C_k(\mathcal{L}),$$

which inherits a well-defined boundary operator from $C_*(\mathcal{K})$, denoted by

$$\overline{\partial}_k : C_k(\mathcal{K},\mathcal{L}) \to C_{k-1}(\mathcal{K},\mathcal{L}).$$

The homology of this quotient chain complex is called the *relative homology*, denoted $H_k(\mathcal{K},\mathcal{L})$. Relative homology captures k-dimensional cycles in $\mathcal{K}$ that become trivial when $\mathcal{L}$ is collapsed or regarded as negligible, and in this way, it isolates the topological features of $\mathcal{K}$ that lie outside $\mathcal{L}$.

3.1.2 Local homology

Let $\mathcal{K}$ be a simplicial complex and v a vertex of $\mathcal{K}$, and let K denote the geometric realization of $\mathcal{K}$.

Definition 3.1. The *local homology* of $\mathcal{K}$ at v in dimension k is defined as

$$H_k^{\mathrm{loc}}(\mathcal{K}, v) := H_k\big(K,\, K \setminus \{v\}\big),$$

i.e., the k-th relative homology group of the pair $(K,\, K \setminus \{v\})$.

Intuitively, $H_k^{\mathrm{loc}}(\mathcal{K}, v)$ measures the homological obstruction to removing the vertex v. Local homology depends only on an arbitrarily small neighborhood of v. Indeed, by the excision theorem, for any open neighborhood U of v in K, there is a canonical isomorphism

$$H_k^{\mathrm{loc}}(\mathcal{K}, v) \cong H_k(U,\, U \setminus \{v\}),$$

hence local homology is a purely local topological invariant.

From a data-analytic viewpoint, $H_k^{\mathrm{loc}}(\mathcal{K}, v)$ characterizes the intrinsic k-dimensional topological structure around v and distinguishes whether v behaves locally like an interior point, a boundary point, or a singular point.

3.1.3 Local Laplacian

Let $\mathcal{K}$ be a simplicial complex and let v be a vertex of $\mathcal{K}$. Denote by $\mathcal{L} = \mathcal{K} \setminus \{v\}$ the maximal subcomplex of $\mathcal{K}$ that does not contain v. Then we obtain a relative chain complex $C_*(\mathcal{K}, \mathcal{L}) = (C_k(\mathcal{K}, \mathcal{L}), \overline{\partial}_k)_{k \geq 0}$, where $\overline{\partial}_k : C_k(\mathcal{K}, \mathcal{L}) \to C_{k-1}(\mathcal{K}, \mathcal{L})$ denotes the relative boundary operator. Furthermore, the relative chain complex $C_*(\mathcal{K}, \mathcal{L})$ admits an induced quotient inner product inherited from the chain complex of $\mathcal{K}$.

Definition 3.2. The k-th *local Laplacian* at vertex v is a self-adjoint operator $\Delta_k^{\mathcal{K},v} : C_k(\mathcal{K}, \mathcal{K} \setminus \{v\}) \to C_k(\mathcal{K}, \mathcal{K} \setminus \{v\})$ defined as

$$\Delta_k^{\mathcal{K},v} := \overline{\partial}_{k+1}\overline{\partial}_{k+1}^* + \overline{\partial}_k^*\overline{\partial}_k, \quad k \geq 0.$$

For $k = 0$, this simplifies to $\Delta_0^{\mathcal{K},v} = \overline{\partial}_1\overline{\partial}_1^*$. Here, $(\cdot)^*$ denotes the adjoint operator with respect to the inner product.

The most important feature of the local Laplacian is its link to local homology.

Theorem 3.3. *Let $\mathcal{K}$ be a finite simplicial complex and $v \in \mathcal{K}$ a vertex. The harmonic space of $\Delta_k^{\mathcal{K},v}$ (its kernel) is equivalent to the local homology group of $\mathcal{K}$ at v, that is,*

$$\ker \Delta_k^{\mathcal{K},v} \cong H_k^{\mathrm{loc}}(\mathcal{K}, v; \mathbb{R}).$$

In addition, every local homology class $[\alpha] \in H_k^{\mathrm{loc}}(\mathcal{K}, v; \mathbb{R})$ has a unique harmonic representative $\gamma \in C_k(\mathcal{K}, \mathcal{K} \setminus \{v\})$ satisfying $\Delta_k^{\mathcal{K},v}\gamma = 0$. This representative is optimal in the sense that it minimizes the L^2-norm among all representatives of the class $[\alpha]$, and is therefore the canonical, smoothest, and most concentrated representative of the corresponding local topological feature.

In practice, the local Laplacian lets us do "microscopic" spectral analysis of $\mathcal{K}$: it turns abstract local topology (like whether v is an interior point, boundary point, or singularity) into computable numbers, making it easy to detect local patterns in high-dimensional data.

3.1.4 Link complex

To describe the local structure explicitly, we introduce the *link* of a vertex: the link of v in $\mathcal{K}$ is the subcomplex

$$\mathrm{Lk}_{\mathcal{K}}(v) = \big\{\tau \in \mathcal{K} \mid v \notin \tau \text{ and } \tau \cup \{v\} \in \mathcal{K}\big\},$$

which records how simplices are attached around v and thus encodes the local connectivity pattern of $\mathcal{K}$ near v. A fundamental result relates local homology to the reduced homology of the link

$$H_k^{\mathrm{loc}}(\mathcal{K}, v) \cong \widetilde{H}_{k-1}\big(\mathrm{Lk}_{\mathcal{K}}(v)\big).$$

Thus, all local topological information at v is encoded in the homology of its link.

A fundamental result relates local homology to the reduced homology of the link

$$H_k^{\mathrm{loc}}(\mathcal{K}, v) \cong \widetilde{H}_{k-1}\big(\mathrm{Lk}_{\mathcal{K}}(v)\big).$$

Thus, all local topological information at v is encoded in the homology of its link. Figure S2 sketches the link complex and the relationship between its reduced homology and the local homology. In particular, $\mathcal{K}$ is locally homeomorphic to a d-dimensional manifold at v if and only if

$$\widetilde{H}_*\big(\mathrm{Lk}_{\mathcal{K}}(v)\big) \cong \widetilde{H}_*(S^{d-1}),$$

meaning that the link has the homology of a $(d-1)$-dimensional sphere.

Consequently, local homology provides a pointwise topological descriptor that is well-suited for detecting boundaries, stratifications, and singularities in point cloud and high-dimensional data.

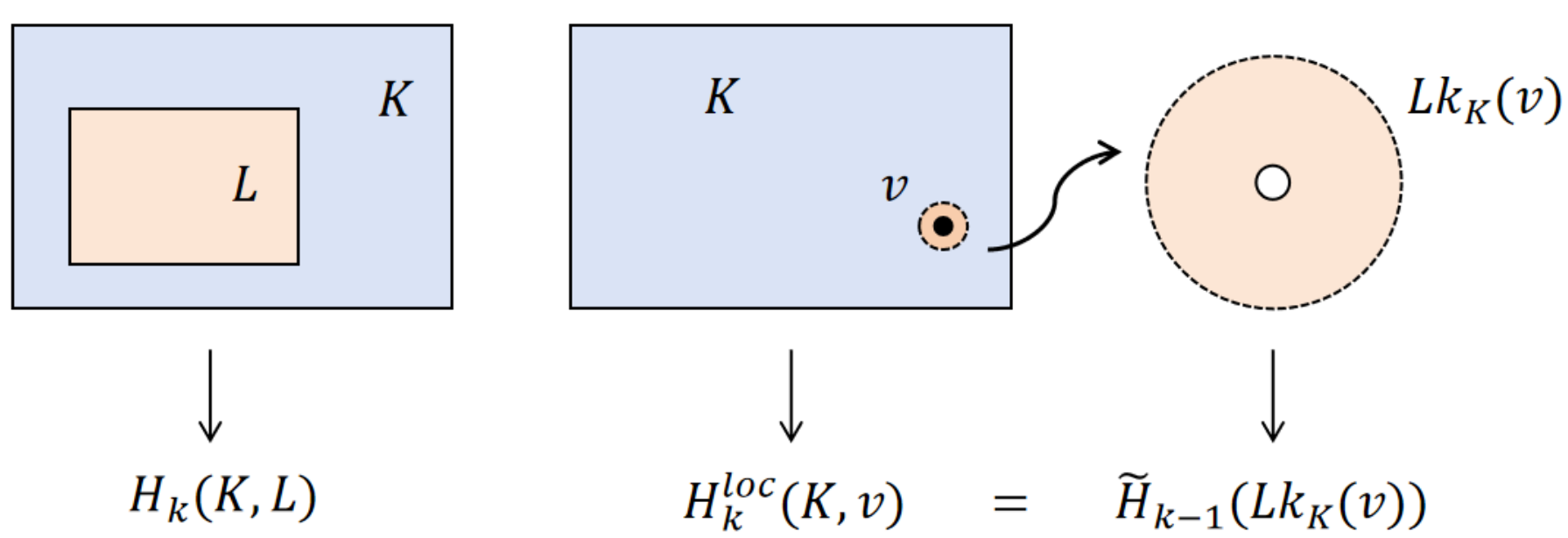

Figure S2: Illustration of relative homology, local homology and the link. The left-hand side illustrates a general relative complex (K, L) and its corresponding relative homology groups. The right-hand side depicts the relative complex of K with respect to a vertex v, whose homology is the local homology at v. This local homology can be characterized in terms of the homology of the link complex of v.

3.1.5 Connection to link Laplacian

As established earlier, the local homology group of $\mathcal{K}$ at vertex v is canonically isomorphic to the reduced homology of the link complex $\mathrm{Lk}_{\mathcal{K}}(v)$, i.e., $H_k^{\mathrm{loc}}(\mathcal{K}, v; \mathbb{R}) \cong \widetilde{H}_{k-1}(\mathrm{Lk}_{\mathcal{K}}(v); \mathbb{R})$. This fundamental isomorphism naturally bridges the local Laplacian $\Delta_k^{\mathcal{K},v}$ to the simplicial Laplacian of the link complex $\mathrm{Lk}_{\mathcal{K}}(v)$, which we refer to as the *link Laplacian*.

First, recall that the link complex $\mathrm{Lk}_{\mathcal{K}}(v)$ consists of all simplices $\tau \in \mathcal{K}$ such that $v \notin \tau$ and $\tau \cup \{v\} \in \mathcal{K}$. The link Laplacian $\Delta_k^{\mathrm{Lk}_{\mathcal{K}}(v)}$ is defined following the standard simplicial Laplacian: for the k-chain group $C_k\left(\mathrm{Lk}_{\mathcal{K}}(v); \mathbb{R}\right)$ equipped with the standard inner product, the link Laplacian is

$$\Delta_k^{\mathrm{Lk}_{\mathcal{K}}(v)} := \partial_{k+1}^v (\partial_{k+1}^v)^* + (\partial_k^v)^* \partial_k^v,$$

where ∂_k^v denotes the boundary operator on $\mathrm{Lk}_{\mathcal{K}}(v)$, and $(\partial_k^v)^*$ is its adjoint operator with respect to the standard inner product on $C_k\left(\mathrm{Lk}_{\mathcal{K}}(v); \mathbb{R}\right)$.

The key connection between the local Laplacian $\Delta_k^{\mathcal{K},v}$ and the link Laplacian $\Delta_{k-1}^{\mathrm{Lk}_{\mathcal{K}}(v)}$ lies in their spectral properties, which are directly linked via the isomorphism $H_k^{\mathrm{loc}}(\mathcal{K}, v) \cong \widetilde{H}_{k-1}\left(\mathrm{Lk}_{\mathcal{K}}(v)\right)$ and the Hodge-theoretic correspondence between harmonic spaces and homology groups:

Theorem 3.4. *Let $\mathcal{K}$ be a finite simplicial complex, and $v \in \mathcal{K}$ a vertex. Then there is a unitary equivalence*

$$\Delta_k^{\mathcal{K},v} \cong \Delta_{k-1}^{\mathrm{Lk}_{\mathcal{K}}(v)}$$

between the local Laplacian and the corresponding link Laplacian at a shifted dimension. In particular, the harmonic space of $\Delta_k^{\mathcal{K},v}$ is isomorphic to the harmonic space of $\Delta_{k-1}^{\mathrm{Lk}_{\mathcal{K}}(v)}$, i.e.,

$$\ker \Delta_k^{\mathcal{K},v} \cong \ker \Delta_{k-1}^{\mathrm{Lk}_{\mathcal{K}}(v)}.$$

In particular, for $k \geq 1$, the local Laplacian can be recovered from the Laplacian of the link. For $k = 0$, the local Laplacian is simply scalar multiplication by $\deg v$, the number of edges incident to vertex v.

The above theorem implies a direct correspondence between the spectral features of the local Laplacian and the link Laplacian. The link Laplacian provides an effective computational tool for the local Laplacian. Although working with quotient relative complexes is often involved, the link complex admits much simpler computations.

3.2 Persistent local spectral analysis

3.2.1 Persistent Local Laplacian

We now extend the notion of local Laplacians to the persistent setting. Let $\mathcal{K}_\bullet = \{\mathcal{K}_t\}_{t=0}^m$ be a filtration of simplicial complexes, and fix a vertex $v \in \mathcal{K}_0$. A filtration $\mathcal{K}_\bullet$ of simplicial complexes is defined as a nested sequence:

$$\mathcal{K}_0 \subseteq \mathcal{K}_1 \subseteq \cdots \subseteq \mathcal{K}_m.$$

Then v is contained in all subsequent complexes of the filtration by definition of a simplicial filtration. This gives rise to a filtration of relative simplicial complexes

$$(\mathcal{K}_0, \mathcal{K}_0 \setminus \{v\}) \hookrightarrow (\mathcal{K}_1, \mathcal{K}_1 \setminus \{v\}) \hookrightarrow \cdots \hookrightarrow (\mathcal{K}_m, \mathcal{K}_m \setminus \{v\}).$$

For any indices $s \leq t$, the inclusion of the underlying simplicial complexes induces an inclusion of relative chain complexes

$$\bar{j}_* : C_*(\mathcal{K}_s, \mathcal{K}_s \setminus \{v\}) \hookrightarrow C_*(\mathcal{K}_t, \mathcal{K}_t \setminus \{v\}).$$

Recall that all relative chain complexes are equipped with the standard inner product induced by the basis of simplices. With respect to this inner product structure, the map $\bar{j}_*$ is an isometric embedding of differential graded inner product spaces.

This chain-level inclusion naturally induces a morphism on local homology groups

$$H_k(\bar{j}_*) : H_k^{\mathrm{loc}}(\mathcal{K}_s, v) \hookrightarrow H_k^{\mathrm{loc}}(\mathcal{K}_t, v), \quad k \geq 0,$$

where $H_k(\mathcal{K}_t, \mathcal{K}_t \setminus \{v\}) = H_k^{\mathrm{loc}}(\mathcal{K}_t, v)$ denotes the k-th local homology group of $\mathcal{K}_t$ at v for any t. We now formalize the notion of persistent local homology:

Definition 3.5 (Persistent Local Homology)**.** For any $s \leq t$, the (s,t)*-persistent local homology* of the filtration $\mathcal{K}_\bullet$ at vertex v is defined as

$$H_k^{s,t}(\mathcal{K}_\bullet; v) := \operatorname{im}\Big(H_k(\bar{j}_*) : H_k^{\mathrm{loc}}(\mathcal{K}_s, v) \hookrightarrow H_k^{\mathrm{loc}}(\mathcal{K}_t, v)\Big), \quad k \geq 0.$$

To connect persistent local homology to harmonic analysis, we introduce the following subspace of the relative chain complex

$$C_k^{s,t}(\mathcal{K}_\bullet; v) = \Big\{x \in C_k(\mathcal{K}_t, \mathcal{K}_t \setminus \{v\}) \mid \overline{\partial}_k^t x \in C_{k-1}(\mathcal{K}_s, \mathcal{K}_s \setminus \{v\})\Big\}.$$

Here, $\overline{\partial}_k^t$ denotes the k-th local boundary operator on $C_k(\mathcal{K}_t, \mathcal{K}_t \setminus \{v\})$. The subspace $C_k^{s,t}(\mathcal{K}_\bullet; v)$ admits a restricted boundary operator

$$\overline{\partial}_k^{s,t} : C_k^{s,t}(\mathcal{K}_\bullet; v) \to C_{k-1}(\mathcal{K}_s, \mathcal{K}_s \setminus \{v\}),$$

where $\overline{\partial}_k^{s,t}$ is simply the restriction of $\overline{\partial}_k^t$ to $C_k^{s,t}(\mathcal{K}_\bullet; v)$. These spaces and operators fit into the following commutative diagram:

$$\begin{array}{ccccc}
C_{k+1}(\mathcal{K}_s, \mathcal{K}_s \setminus \{v\}) & \xrightarrow{\overline{\partial}_{k+1}^s} & C_k(\mathcal{K}_s, \mathcal{K}_s \setminus \{v\}) & \xrightarrow{\overline{\partial}_k^s} & C_{k-1}(\mathcal{K}_s, \mathcal{K}_s \setminus \{v\}) \\
\big\downarrow & & \big\| & & \big\| \\
C_{k+1}^{s,t}(\mathcal{K}_\bullet; v) & \xrightarrow{\overline{\partial}_{k+1}^{s,t}} & C_k(\mathcal{K}_s, \mathcal{K}_s \setminus \{v\}) & \xrightarrow{\overline{\partial}_k^s} & C_{k-1}(\mathcal{K}_s, \mathcal{K}_s \setminus \{v\}) \\
\big\downarrow & & \big\downarrow & & \big\downarrow \\
C_{k+1}(\mathcal{K}_t, \mathcal{K}_t \setminus \{v\}) & \xrightarrow{\overline{\partial}_{k+1}^t} & C_k(\mathcal{K}_t, \mathcal{K}_t \setminus \{v\}) & \xrightarrow{\overline{\partial}_k^t} & C_{k-1}(\mathcal{K}_t, \mathcal{K}_t \setminus \{v\}).
\end{array}$$

Definition 3.6 (Persistent Local Laplacian)**.** For any $s \leq t$, the k-th (s,t)*-persistent local Laplacian* $\Delta_k^{s,t}(\mathcal{K}_\bullet; v) : C_k(\mathcal{K}_s, \mathcal{K}_s \setminus \{v\}) \to C_k(\mathcal{K}_s, \mathcal{K}_s \setminus \{v\})$ of the filtration $\mathcal{K}_\bullet$ at vertex v is the operator

$$\Delta_k^{s,t}(\mathcal{K}_\bullet; v) := (\overline{\partial}_k^s)^* \overline{\partial}_k^s + \overline{\partial}_{k+1}^{s,t} (\overline{\partial}_{k+1}^{s,t})^*, \quad k \geq 0.$$

Analogous to the non-persistent case, the persistent local Laplacian $\Delta_k^{s,t}(\mathcal{K}_\bullet; v)$ is a self-adjoint, positive semi-definite operator. The kernel of this operator, the space of harmonic vectors with respect to $\Delta_k^{s,t}(\mathcal{K}_\bullet; v)$, is called the (s,t)-persistent local harmonic space, denoted $\mathcal{H}_k^{s,t}(\mathcal{K}_\bullet; v)$.

The key connection between persistent local harmonic spaces and persistent local homology is given by the following theorem:

Theorem 3.7. *For any $s \leq t$ and integer $k \geq 0$, there is a canonical isomorphism of linear spaces*

$$\mathcal{H}_k^{s,t}(\mathcal{K}_\bullet; v) \cong H_k^{s,t}(\mathcal{K}_\bullet; v).$$

Here, $H_k^{s,t}(\mathcal{K}_\bullet; v)$ denotes the (s,t)-persistent local homology group.

3.2.2 Persistent Link Laplacian

Recall from the previous subsection that for any simplicial complex $\mathcal{K}$ and a vertex $v \in \mathcal{K}$, the link complex $\mathrm{Lk}_{\mathcal{K}}(v)$ can be constructed, which serves as a key tool for computing the local Laplacian of $\mathcal{K}$ at v. Extending this construction to the persistent setting, we first derive a filtration of link complexes from a given simplicial filtration.

Let $\mathcal{K}_\bullet = \{\mathcal{K}_t\}_{t=0}^m$ be a filtration of simplicial complexes, and fix a vertex $v \in \mathcal{K}_0$. For each t, the link complex $\mathrm{Lk}_{\mathcal{K}_t}(v)$ is a subcomplex of $\mathrm{Lk}_{\mathcal{K}_{t+1}}(v)$. This gives rise to a filtration of link complexes:

$$\mathrm{Lk}_{\mathcal{K}_0}(v) \hookrightarrow \mathrm{Lk}_{\mathcal{K}_1}(v) \hookrightarrow \cdots \hookrightarrow \mathrm{Lk}_{\mathcal{K}_m}(v).$$

We denote this link filtration by $\mathrm{Lk}_{\mathcal{K}_\bullet}(v) = \{\mathrm{Lk}_{\mathcal{K}_t}(v)\}_{t=0}^m$. Since $\mathrm{Lk}_{\mathcal{K}_\bullet}(v)$ is a filtration of simplicial complexes, we can define the standard persistent Laplacian on this filtration, following the same construction as the persistent Laplacian. We denote this persistent Laplacian by $\Delta_k^{s,t}(\mathrm{Lk}_{\mathcal{K}_\bullet}(v))$, where $s \leq t$ and $k \geq 0$ denote the persistence interval and homological dimension, respectively.

The core result is a fundamental relationship between the persistent local Laplacian and the persistent link Laplacian, which generalizes the non-persistent connection between local and link Laplacians.

Theorem 3.8. *Let $\mathcal{K}_\bullet = \{\mathcal{K}_t\}_{t=0}^m$ be a filtration of simplicial complexes, and fix a vertex $v \in \mathcal{K}_0$. For any indices $s \leq t$ and integer $k \geq 0$, the (s,t)-persistent local Laplacian $\Delta_k^{s,t}(\mathcal{K}_\bullet; v)$ is unitarily equivalent to the (s,t)-persistent Laplacian of the link filtration $\mathrm{Lk}_{\mathcal{K}_\bullet}(v)$ at dimension $k-1$, i.e.,*

$$\Delta_k^{s,t}(\mathcal{K}_\bullet; v) \cong \Delta_{k-1}^{s,t}(\mathrm{Lk}_{\mathcal{K}_\bullet}(v)).$$

As a direct consequence, their kernels (persistent harmonic spaces) are isomorphic

$$\ker \Delta_k^{s,t}(\mathcal{K}_\bullet; v) \cong \ker \Delta_{k-1}^{s,t}(\mathrm{Lk}_{\mathcal{K}_\bullet}(v)).$$

This unitary equivalence implies that the persistent local Laplacian $\Delta_k^{s,t}(\mathcal{K}_\bullet; v)$ and the persistent link Laplacian $\Delta_{k-1}^{s,t}(\mathrm{Lk}_{\mathcal{K}_\bullet}(v))$ have identical spectral properties. In particular, their persistent harmonic spaces encode the same algebraic information, which bridges the persistent local homology of $\mathcal{K}_\bullet$ at v and the persistent homology of the link filtration $\mathrm{Lk}_{\mathcal{K}_\bullet}(v)$.

3.3 Computational complexity analysis

This paper focuses only on two core parts of the computational complexity of the persistent Laplacian for discussion: first, the computational complexity of constructing the Laplacian matrix itself; second, the computational complexity of solving all eigenvalues of the Laplacian matrix.

It should be noted that in practical computing scenarios, there are often various optimization algorithms for specific cases such as efficient implementation for sparse matrices, targeted algorithms that only solve the smallest positive eigenvalue (instead of all eigenvalues), etc. Such optimizations will significantly reduce the actual computational complexity. In addition, there may be other simpler algorithmic ideas to further improve computational efficiency.

The discussions in this paper are all based on the mathematical algorithm level and limited to the premise of solving for exact numerical values, without involving various optimization strategies in engineering implementation, and only analyzing the computational complexity at the theoretical level.

3.3.1 Computational complexity of Laplacian matrix

In practical applications, the topological multi-scale features that we commonly compute are not the persistent Laplacian itself, but rather the Laplacian corresponding to each filtration parameter t. As a result, the Laplacian Δ_k^t becomes a parameter-dependent operator with respect to t. Therefore, the computational complexity of computing the Laplacian can be reduced to that of calculating the Laplacian Δ_k^t for a fixed filtration parameter t. Furthermore, our analysis focuses on the computation of 0- and 1-dimensional Laplacians, as these are the dimensions relevant to practical application scenarios.

We begin by introducing the basic notation used in the complexity analysis.

- $\mathcal{K}$: a finite simplicial complex;
- n_0, n_1, n_2: the numbers of vertices (0-simplices), edges (1-simplices), and triangles (2-simplices) in $\mathcal{K}$, respectively;
- $\mathbf{D}_1$: the matrix representation of the boundary operator ∂_1, where $\mathbf{D}_1 \in \mathbb{R}^{n_0 \times n_1}$, each column corresponds to an edge and the nonzero entries are ± 1;
- $\mathbf{D}_2$: the matrix representation of the boundary operator ∂_2, where $\mathbf{D}_2 \in \mathbb{R}^{n_1 \times n_2}$, each column corresponds to a triangle and the nonzero entries are ± 1;
- $\mathbf{L}_0$: the 0-dimensional Laplacian matrix, corresponding to the operator $\Delta_0 = \partial_1 \partial_1^*$, where
$$\mathbf{L}_0 = \mathbf{D}_1 \mathbf{D}_1^T \in \mathbb{R}^{n_0 \times n_0};$$
- $\mathbf{L}_1$: the 1-dimensional Laplacian matrix, corresponding to the operator $\Delta_1 = \partial_1^* \partial_1 + \partial_2 \partial_2^*$, where
$$\mathbf{L}_1 = \mathbf{D}_1^T \mathbf{D}_1 + \mathbf{D}_2 \mathbf{D}_2^T \in \mathbb{R}^{n_1 \times n_1}.$$

The computational complexity of constructing the Laplacian matrices is analyzed as follows. We first consider the construction of the 0-dimensional Laplacian matrix $\mathbf{L}_0$, which does not involve 2-simplices. The first step is to construct the 1-boundary matrix $\mathbf{D}_1$ by traversing all n_1 edges and inserting the corresponding nonzero entries ± 1, which requires $O(n_1)$ time. The second step computes the matrix product $\mathbf{L}_0 = \mathbf{D}_1 \mathbf{D}_1^T$, which requires $O(n_0^2 n_1)$ time using standard dense matrix multiplication. Combining these steps, the total computational complexity for constructing $\mathbf{L}_0$ is $O(n_1) + O(n_0^2 n_1)$, which simplifies to $O(n_0^2 n_1)$ under the dense matrix assumption.

For the construction of the 1-dimensional Laplacian matrix $\mathbf{L}_1$, the presence of 2-simplices contributes to the computational complexity. The first step constructs the boundary matrices $\mathbf{D}_1$ and $\mathbf{D}_2$: constructing $\mathbf{D}_1$ requires $O(n_1)$ time, while constructing $\mathbf{D}_2$ requires traversing all n_2 triangles, each corresponding to a column of $\mathbf{D}_2$ with three nonzero entries ± 1, resulting in a complexity of $O(n_2)$. The second step computes $\mathbf{D}_1^T \mathbf{D}_1$ using standard dense matrix multiplication, which requires $O(n_1^2 n_0)$ time. The third step computes $\mathbf{D}_2 \mathbf{D}_2^T$ using dense matrix multiplication with a complexity of $O(n_1^2 n_2)$. The fourth step performs the matrix addition $\mathbf{L}_1 = \mathbf{D}_1^T \mathbf{D}_1 + \mathbf{D}_2 \mathbf{D}_2^T$, which requires $O(n_1^2)$ time due to element-wise addition. Summing these steps, the total complexity for constructing $\mathbf{L}_1$ is $O(n_1 + n_2) + O(n_1^2 n_0) + O(n_1^2 n_2) + O(n_1^2)$, and the dominant terms from the matrix multiplications yield an overall complexity of $O(n_1^2(n_0 + n_2))$.

Suppose each vertex of $\mathcal{K}$ is contained in at most q simplices of $\mathcal{K}$. We first compute the link complex $\mathrm{Lk}(\mathcal{K}, v)$ for every vertex $v \in \mathcal{K}$. Since each vertex is contained in at most q simplices, constructing the link complex of a single vertex requires processing at most q associated simplices. Therefore, the link complexes of all vertices can be constructed in $O(n_0 q)$ time.

Based on these link complexes, we construct the 0-, 1-, and 2-dimensional combinatorial Laplacian matrices for each $\mathrm{Lk}(\mathcal{K}, v)$. As the simplicial complexes under consideration have dimension at most 2, the numbers of vertices, edges, and triangles in each link complex are all bounded by q, and hence the total number of simplices in each link complex is $O(q)$. The construction time of the combinatorial Laplacian matrix in each dimension is linear in the number of simplices of the corresponding dimension. Consequently, the 0-, 1-, and 2-dimensional combinatorial Laplacian matrices for a single link complex can be computed in $O(q)$ time. Moreover, by traversing the link complexes for all vertices v, the total computational complexity is $O(qn_0)$.

3.3.2 Spectra calculation complexity of Laplacian matrices

Beyond the construction of Laplacian matrices, the computational complexity of solving their spectra (i.e., all eigenvalues) is a critical metric for practical topological feature extraction. We analyze this complexity based on the QR algorithm, a standard method for exact computation of all eigenvalues of real symmetric matrices, which is widely adopted in theoretical complexity analysis for numerical linear algebra.

For the conventional global Laplacians, the spectra calculation complexity is dominated by the size of the Laplacian matrix. The 0-dimensional Laplacian matrix $\mathbf{L}_0$ is an $n_0 \times n_0$ real symmetric matrix, and the QR algorithm for solving all its eigenvalues has a time complexity of $O(n_0^3)$. This cubic complexity arises from the iterative orthogonalization steps in the QR algorithm, which scale with the cube of the matrix dimension for dense symmetric matrices. For the 1-dimensional global Laplacian matrix $\mathbf{L}_1$ (an $n_1 \times n_1$ real symmetric positive semi-definite matrix), the spectra calculation complexity is $O(n_1^3)$, as the matrix size (rather than the presence of 2-simplices) determines the computational cost of eigenvalue decomposition.

In contrast, the spectra calculation complexity of local Laplacians (based on link complexes) is drastically reduced due to the bounded size of link complexes. As each vertex $v \in \mathcal{K}$ is contained in at most q simplices, the link complex $\mathrm{Lk}(\mathcal{K}, v)$ has a maximum size of q (i.e., the number of vertices/edges/triangles in $\mathrm{Lk}(\mathcal{K}, v)$ is bounded by q). Thus, the local Laplacian matrix for a single link complex is at most a $q \times q$ matrix, and its spectra can be computed in $O(q^3)$ time using the same QR algorithm. When extending to all vertices in $\mathcal{K}$, the total complexity for spectra calculation of local Laplacians is $O(n_0 q^3)$, where n_0 accounts for the traversal over all vertices.

Table S1: Computational complexity of Laplacian matrix and spectra calculation

**Laplacian Type**	**Laplacian complexity**	**Spectra complexity**	**Total Complexity**
**Combinatorial (Global) Laplacian**			
0-dimensional ($\mathbf{L}_0$)	$O(n_0^2 n_1)$	$O(n_0^3)$	$O(n_0^2 n_1 + n_0^3)$
1-dimensional ($\mathbf{L}_1$)	$O(n_1^2(n_0 + n_2))$	$O(n_1^3)$	$O(n_1^2(n_0 + n_2) + n_1^3)$
**Local Laplacian (Based on Link Complexes)**			
Link complex	$O(n_0 q)$	–	$O(n_0 q)$
0/1/2-dim link Laplacian	$O(q)$	$O(q^3)$	$O(q + q^3)$
Total Local Laplacian	$O(n_0 q)$	$O(n_0 q^3)$	$O(n_0 q + n_0 q^3)$

The key advantage of local Laplacians becomes evident when considering large-scale systems where $n_0, n_1, n_2 \gg q$. In such cases, $O(n_0 q^3)$ is significantly smaller than the global complexity $O(n_0^3)$ or $O(n_1^3)$, as q is a small constant (the maximum number of simplices containing a single vertex) while n_0/n_1 scale with the size of the entire simplicial complex. This reduction in complexity

validates the practicality of local Laplacians for topological analysis of large-scale datasets.

4 Result analysis of PLL on protein-ligand binding affinity

4.1 Comparisons of different model strategies.

The modeling performance comparison of different strategies on the test sets of the three PDBbind benchmark datasets are given in Table S2.

Dataset	PLL	PLL/lig	Transformer	PLL+ Transformer
PDBbind-v2007	0.813(1.971)	0.811(1.979)	0.785(2.006)	**0.827 (1.925)**
PDBbind-v2013	0.792(1.976)	0.789(1.988)	0.791(1.974)	**0.813 (1.932)**
PDBbind-v2016	0.850(1.672)	0.844(1.690)	0.836(1.713)	**0.861 (1.646)**
Average	0.818 (1.873)	0.818 (1.873)	0.804 (1.898)	**0.834 (1.834)**

Table S2: Modeling performance of different strategies on the test sets of the three PDBbind benchmark datasets. The evaluation metrics are the Pearson correlation coefficient (R) and the root-mean-square error (RMSE, in kcal/mol, in the parenthesis). Each model was trained and evaluated over ten independent runs using different random seeds, and the reported results are the averaged metric values. PLL denotes the machine learning models with persistent local Laplacian characterization of protein-ligand complex and the ligand molecule. PLL/lig denotes model with the persistent local Laplacian characterization solely on protein-ligand complexes. Transformer refers to sequence-based models using natural language processing. PLL + Transformer represents the consensus predictions obtained by averaging the outputs of the PLL and Transformer models, which is used as our final model referred to as PLLML.

4.2 Impacts of filtration ranges on PLL models.

We are interested in the impacts of varying the upper bound of the filtration radius on predictive performance of our persistent local Laplacian models. To identify an effective filtration range, we evaluated model performance at different filtration radii on three PDBbind benchmark datasets. We considered two types of models: one using molecular features derived solely from the protein–ligand complex, and another incorporating additional ligand-specific molecular features. For both models, the optimal performance is observed at upper filtration scale of 6Å on PDBbind-v2007 and PDBbind-v2016 dataset, while the optimal is at 7Å on PDBbind-v2013 dataset. The details are provided in Table S3 and Table S4. The data visualization is given in Figure 6 of the main text.

4.3 Computational efficiency comparisons between PLL and persistent Laplacian

We considered several protein–ligand complexes to compare the computational efficiency of the persistent local Laplacian and persistent Laplacian methods. Specifically, we selected ten large protein–ligand complexes from the PDBbind-v2016 dataset. A cutoff distance of 12 Å from the ligand was used to select protein atoms for the topological characterization of protein–ligand binding interactions. The second column in Table S5 indicates the number of filtered atoms after applying cutoff distance for the ten proteins.

For both methods, we designed molecular features using an element-specific strategy. For the persistent local Laplacian, we used the same featurization parameter settings as those described in

Table S3: Effect of filtration radius on PLL predictive performance. Values are reported as mean (standard deviation) over 20 runs with different random seeds.

Metric	4Å	5Å	6Å	7Å	8Å	9Å	10Å
				**PDBbind-v2007**			
PCC	0.794 (0.002)	0.807 (0.002)	0.811 (0.002)	0.810 (0.001)	0.808 (0.002)	0.808 (0.001)	0.808 (0.001)
RMSE	2.053 (0.006)	1.995 (0.006)	1.979 (0.005)	1.965 (0.005)	1.968 (0.007)	1.966 (0.005)	1.960 (0.005)
				**PDBbind-v2013**			
PCC	0.777 (0.002)	0.787 (0.002)	0.789 (0.001)	0.790 (0.002)	0.789 (0.002)	0.786 (0.002)	0.786 (0.002)
RMSE	2.023 (0.004)	1.994 (0.004)	1.988 (0.004)	1.984 (0.006)	1.986 (0.005)	1.993 (0.005)	1.994 (0.005)
				**PDBbind-v2016**			
PCC	0.834 (0.001)	0.841 (0.001)	0.844 (0.001)	0.844 (0.002)	0.842 (0.001)	0.841 (0.001)	0.839 (0.001)
RMSE	1.731 (0.004)	1.704 (0.004)	1.690 (0.004)	1.689 (0.006)	1.693 (0.003)	1.697 (0.005)	1.700 (0.004)

Table S4: Effect of filtration radius on PLL predictive performance. Values are reported as mean (standard deviation) over 20 runs.

Metric	4Å	5Å	6Å	7Å	8Å	9Å	10Å
				**PDBbind-v2007**			
PCC	0.800 (0.002)	0.811 (0.001)	0.813 (0.001)	0.815 (0.002)	0.812 (0.001)	0.811 (0.001)	0.813 (0.002)
RMSE	2.047 (0.006)	1.997 (0.005)	1.975 (0.005)	1.959 (0.006)	1.963 (0.004)	1.959 (0.004)	1.951 (0.006)
				**PDBbind-v2013**			
PCC	0.788 (0.002)	0.791 (0.001)	0.794 (0.001)	0.793 (0.002)	0.791 (0.001)	0.790 (0.001)	0.789 (0.001)
RMSE	1.999 (0.006)	1.988 (0.004)	1.980 (0.004)	1.979 (0.005)	1.981 (0.004)	1.985 (0.004)	1.986 (0.004)
				**PDBbind-v2016**			
PCC	0.841 (0.001)	0.845 (0.001)	0.850 (0.001)	0.848 (0.001)	0.846 (0.001)	0.845 (0.001)	0.842 (0.001)
RMSE	1.719 (0.004)	1.702 (0.004)	1.672 (0.004)	1.684 (0.005)	1.684 (0.005)	1.686 (0.004)	1.692 (0.003)

the manuscript. For the persistent Laplacian, we considered the 0-th Laplacians and extracted nine statistical summaries of their nonzero eigenvalues, including the sum, mean, median, maximum, standard deviation, variance, minimum, and the squared L_2 norm, together with the number of zero eigenvalues. The persistent Laplacian has been employed in our previous investigations [8]. The PerSpect model [9] was the first to incorporate the persistent Laplacian into protein–ligand binding affinity prediction. Here, we adopted a similar persistent Laplacian feature design strategy to that used in PerSpect [9]. A filtration radius ranging from 0 to 12 Å with a step size of 0.1 Å was used.

The table presents a comparison of the CPU time required by the two methods to generate molecular features. As shown in the table, the persistent local Laplacian requires less CPU time than the persistent Laplacian for molecular feature generation. Moreover, the computational cost of the persistent local Laplacian can be further reduced through parallel computation because the local Laplacian matrices associated with different atoms can be generated independently. On average, the feature generation of persistent local Laplacian take 3 times of that by persistent Laplacian across the 10 large proteins. These results demonstrate the computational efficiency of the persistent local Laplacian compared with the persistent Laplacian.

Table S5: The CPU time (in seconds) comparisons between persistent local Laplacian (PLL) and persistent Laplacian (PL) across several large proteins.

PDBID	atom numbers within cutoff distance	CPU(s)-PL	CPU(s)-PLL
3drg	1543	207	64
1fo0	1420	160	57
2xef	1314	142	56
3drf	1254	110	42
4ca5	1245	134	55
2xyd	1242	133	51
2xy9	1241	126	54
1i5r	1232	107	43
3bzf	1228	133	50
4ca8	1216	117	48

5 Molecular large language models

5.1 Transformer-based protein language model

Transformer-based protein large language models, such as ESM (Evolutionary Scale Modeling) [10], are trained on hundreds of millions of protein sequences and have demonstrated outstanding performance across a wide range of protein analysis tasks. In this work, we use ESM-2 to generate protein sequence embeddings, specifically the esm2_t33_650M_UR50D model. ESM-2 employs a deep architecture with 34 layers and 650 million parameters, where each layer encodes input tokens into 1280-dimensional vectors. For every input protein sequence, we extract the representations from the final layer and compute the mean across all sequence positions, yielding a single 1280-dimensional embedding vector. We use this vector as protein sequence embeddings in our machine learning modeling.

5.2 Transformer-based small molecular language model

A Transformer-based deep learning framework was developed to extract molecular representations from SMILES strings [11], and it has proven to be a powerful tool for machine learning applications involving small molecules [12]. This model was pretrained on more than 700 million SMILES strings collected from databases such as ChEMBL, PubChem, and ZINC. Three pretrained variants were released—model-C, model-CP, and model-CPZ. In this study, we use model-CPZ to generate molecular descriptors for ligands. For each ligand, the model outputs a 256×512 matrix, where 256 corresponds to the sequence tokens representing the molecule and 512 is the embedding dimension for each token. The final ligand descriptor is obtained by taking the first embedding vector among the 256 token embeddings, yielding a fixed-length feature vector.

6 Evaluation metrics

To rigorously assess the predictive accuracy of our binding affinity models, we use two standard evaluation indicators: the Pearson correlation coefficient (PCC) and the root mean squared error (RMSE). The PCC measures the linear correlation between experimental and predicted values and is defined as

$$\text{PCC} = \frac{\sum_{m=1}^{M}(y_m^e - \bar{y}^e)(y_m^p - \bar{y}^p)}{\sqrt{\sum_{m=1}^{M}(y_m^e - \bar{y}^e)^2}\sqrt{\sum_{m=1}^{M}(y_m^p - \bar{y}^p)^2}},$$

where y_m^e and y_m^p denote the experimental and predicted affinities for the m-th complex, and $\bar{y}^e$ and $\bar{y}^p$ are their respective means.

The RMSE quantifies the average prediction error and is computed as

$$\text{RMSE} = \sqrt{\frac{1}{M}\sum_{m=1}^{M}(y_m^e - y_m^p)^2}.$$

These two metrics are used to benchmark model performance on the three PDBbind datasets. The original labels, provided in pK_d, are converted to binding free energies ΔG (in kcal/mol) by multiplying by a constant factor of 1.3633 [13].

References

[1] Herbert Edelsbrunner and John Harer. Persistent homology-a survey. *Contemporary mathematics*, 453(26):257–282, 2008.

[2] Afra Zomorodian and Gunnar Carlsson. Computing persistent homology. *Discrete & Computational Geometry*, 33(2):249–274, 2004.

[3] Jiahui Chen, Rundong Zhao, Yiying Tong, and Guo-Wei Wei. Evolutionary de rham-hodge method. *Discrete and continuous dynamical systems. Series B*, 26(7):3785, 2021.

[4] Facundo Mémoli, Zhengchao Wan, and Yusu Wang. Persistent laplacians: Properties, algorithms and implications. *SIAM Journal on Mathematics of Data Science*, 4(2):858–884, 2022.

[5] Rui Wang, Duc Duy Nguyen, and Guo-Wei Wei. Persistent spectral graph. *International journal for numerical methods in biomedical engineering*, 36(9):e3376, 2020.

[6] Jian Liu, Hongsong Feng, and Kefeng Liu. Local laplacian: theory and models for data analysis. *arXiv preprint arXiv:2603.07591*, 2026.

[7] Jian Liu, Jingyan Li, and Jie Wu. The algebraic stability for persistent Laplacians. *Homology, Homotopy and Applications*, 26(2):297–323, 2024.

[8] Hongsong Feng and Guo-Wei Wei. Virtual screening of drugbank database for herg blockers using topological laplacian-assisted ai models. *Computers in biology and medicine*, 153:106491, 2023.

[9] Zhenyu Meng and Kelin Xia. Persistent spectral–based machine learning (perspect ml) for protein-ligand binding affinity prediction. *Science advances*, 7(19):eabc5329, 2021.

[10] Alexander Rives, Joshua Meier, Tom Sercu, Siddharth Goyal, Zeming Lin, Jason Liu, Demi Guo, Myle Ott, C Lawrence Zitnick, Jerry Ma, et al. Biological structure and function emerge from scaling unsupervised learning to 250 million protein sequences. *Proceedings of the National Academy of Sciences*, 118(15):e2016239118, 2021.

[11] Dong Chen, Jiaxin Zheng, Guo-Wei Wei, and Feng Pan. Extracting predictive representations from hundreds of millions of molecules. *The journal of physical chemistry letters*, 12(44):10793–10801, 2021.

[12] Li Shen, Hongsong Feng, Yuchi Qiu, and Guo-Wei Wei. SVSBI: sequence-based virtual screening of biomolecular interactions. *Communications Biology*, 6(1):536, 2023.

[13] Hongsong Feng, Li Shen, Jian Liu, and Guo-Wei Wei. Mayer-homology learning prediction of protein-ligand binding affinities. *Journal of computational biophysics and chemistry*, 24(02):253–266, 2025.
